# Gallic Acid Ameliorated Impaired Glucose and Lipid Homeostasis in High Fat Diet-Induced NAFLD Mice

**DOI:** 10.1371/journal.pone.0096969

**Published:** 2014-06-11

**Authors:** Jung Chao, Teh-Ia Huo, Hao-Yuan Cheng, Jen-Chieh Tsai, Jiunn-Wang Liao, Meng-Shiou Lee, Xue-Mei Qin, Ming-Tsuen Hsieh, Li-Heng Pao, Wen-Huang Peng

**Affiliations:** 1 Institute of Pharmacology, College of Medicine, National Yang-Ming University, Taipei, Taiwan; 2 Department of Oncology and Internal Medicine, National Taiwan University Hospital, Taipei, Taiwan; 3 Department of Nursing, Chung Jen College of Nursing, Health Sciences and Management, Chia-Yi, Taiwan; 4 Department of Health and Nutrition Biotechnology, College of Health Science, Asia University, Taichung, Taiwan; 5 Jen-Teh Junior College of Medicine, Nursing and Management, Miaoli, Taiwan; 6 Graduate Institute of Veterinary Pathology, National Chung Hsing University, Taichung, Taiwan; 7 Department of Chinese Pharmaceutical Sciences and Chinese Medicine Resources, College of Pharmacy, China Medical University, Taichung, Taiwan; 8 Modern Research Center for Traditional Chinese Medicine of Shanxi University, Taiyuan, China; 9 Research Center for Industry of Human Ecology, Chang Gung University of Science and Technology, Taoyuan, Taiwan; 10 School of Pharmacy, National Defense Medical Center, Taipei, Taiwan; University of Cordoba, Spain

## Abstract

Gallic acid (GA), a naturally abundant plant phenolic compound in vegetables and fruits, has been shown to have potent anti-oxidative and anti-obesity activity. However, the effects of GA on nonalcoholic fatty liver disease (NAFLD) are poorly understood. In this study, we investigated the beneficial effects of GA administration on nutritional hepatosteatosis model by a more “holistic view” approach, namely ^1^H NMR-based metabolomics, in order to prove efficacy and to obtain information that might lead to a better understanding of the mode of action of GA. Male C57BL/6 mice were placed for 16 weeks on either a normal chow diet, a high fat diet (HFD, 60%), or a high fat diet supplemented with GA (50 and 100 mg/kg/day, orally). Liver histopathology and serum biochemical examinations indicated that the daily administration of GA protects against hepatic steatosis, obesity, hypercholesterolemia, and insulin resistance among the HFD-induced NAFLD mice. In addition, partial least squares discriminant analysis scores plots demonstrated that the cluster of HFD fed mice is clearly separated from the normal group mice plots, indicating that the metabolic characteristics of these two groups are distinctively different. Specifically, the GA-treated mice are located closer to the normal group of mice, indicating that the HFD-induced disturbances to the metabolic profile were partially reversed by GA treatment. Our results show that the hepatoprotective effect of GA occurs in part through a reversing of the HFD caused disturbances to a range of metabolic pathways, including lipid metabolism, glucose metabolism (glycolysis and gluconeogenesis), amino acids metabolism, choline metabolism and gut-microbiota-associated metabolism. Taken together, this study suggested that a ^1^H NMR-based metabolomics approach is a useful platform for natural product functional evaluation. The selected metabolites are potentially useful as preventive action biomarkers and could also be used to help our further understanding of the effect of GA in hepatosteatosis mice.

## Introduction

Nonalcoholic fatty liver disease (NAFLD) is a slowly progressive affliction that includes a wide spectrum of liver diseases, ranging from simple fatty liver to nonalcoholic steatohepatitis (NASH); these may eventually progress to liver cirrhosis, and hepatocellular carcinoma [1]. As a primary cause of abnormal liver function tests in Asia over the last few years, NAFLD has become an important clinical issue. However, effective therapies for treating NAFLD have yet to be found [2] and this has contributed to an increased use by sufferers of natural products.

Plant-derived polyphenol compounds possess a wide range of pharmacological properties and their action has been the subject of considerable interest in recent years. Gallic acid (GA), an endogenous plant phenol, is a naturally abundant plant compound in vegetables, tea, grapes, berries, as well as wine [3]–[9]. GA have been reported to have potent free radical scavenging and anti-oxidative activities [10], [11] and therefore the study of the mechanism of action of GA has received much attention recently. Many GA-rich plants exhibit protective effects against liver injury [3]–[5]. In addition, GA seems to have a variety of different pharmacological activities, including anti-inflammatory [9], [12], anti-obesity [8], [10], [11], and anti-cancer activities [13]. Furthermore, the protective effect of GA on hepatic lipid peroxide metabolism, glycoprotein components and lipid peroxidation in the STZ-induced diabetic rats has been reported [14]. The previous subchronic toxicology study has suggested that GA is safe and seems to have a no-observed-adverse-effect level (NOAEL) at doses of 119 and 128 mg/kg/day, respectively for male and female rats [15]. Even though many reports have revealed that GA seems to play an important role in the prevention of diabetes and metabolic disease development, direct evidence of these effects and the mechanism underlying the action of GA on NAFLD remain unclear.

Metabolomics is defined as the quantitative measurement of the time-related multiparametric metabolic responses of multicellular systems to pathophysiological stimuli or a genetic modification [16]. The metabolomics approach has demonstrated potential in many fields, including disease diagnosis [17]–[19], investigations of toxicological mechanisms [20], [21], plant metabolomics [22], [23], determination of the mechanism of drug treatment and assessing the effect of nutritional intervention [19], [24]–[28]. The usually used analytical techniques of metabolomics can be classified into mass spectrometry (MS)-based detection methods and nuclear magnetic resonance (NMR)-based detection methods [29]. ^1^H NMR has been used as a major analytical tool for many applications, because one of the major advantages of NMR is that the biological fluid does not require any physical or chemical treatment before the analysis [29]. In addition, NMR is a very useful technique for structure elucidation using various two-dimensional NMR measurements without the further fractionation of the biological samples [22], [30].

It is rational to propose that when trying to elucidate the preventive effects and mechanisms of GA on NAFLD, the use of these results is likely to provide strong evidence in support of, at least a part, the preventive effects on the metabolic diseases of this functional food when used daily. The aim of this study is to investigate the beneficial effects of GA on nutritional hepatosteatosis by ^1^H NMR-based metabolomics using an animal model. The mechanisms by which GA affects the mice were elucidated from a global perspective and used a metabolomics approach to explore the complicated systematic changes that occur in HFD-induced nutritional steatosis model mouse serum and urine samples. The experiment results and proposed pathways will help to elucidate the multiple targets involved in the hepatoprotective activities of GA.

## Materials and Methods

### Chemicals and Reagents

Gallic acid (98%), D_2_O (99.9%), and chloroform-*d* containing tetramethylsilane (TMS) (99.9%) were purchased from Sigma-Aldrich (St. Louis, MO). Trimethylsilane propionic acid sodium salt (TSP) was purchased from Merck (Darmstadt, Germany).

### Animals Treatment and Sample Collection

Male 10-week old C57BL/6 mice were purchased from BioLASCO Taiwan Co., Ltd. All mice were housed alone in standard cages for one week at least before the experiments began The animals were kept at a constant temperature of 22±1°C, relative humidity of 55±5% and under a 12 h light–dark cycle (08∶00 to 20∶00). They had free access to food and water. The animals were divided into three groups (**Figure S1A**): (1) a normal chow diet (normal group), n = 10, (2) a high fat diet (HFD group) n = 11, and (3) a high fat diet treated with GA (treatment group, high fat diet+GA 50 and 100 mg/kg/day, orally), n = 10. The high fat diet consisted of food with 60% of the calories coming from fat (5.24 kcal/g, 60% kcal from lard/soybean 9.8∶1, D12492; Research Diets, New Brunswick NJ) (**Table S1** and **File S1**). This diet has previously been demonstrated to induce obesity in C57BL/6 mice [31]. The normal chow diet consisted of food with 12.7% of the calories coming from fat (4.14 kcal/g, LabDiet 5010 Rodent Diet, Richmond, IN, USA).

The normal and HFD groups of mice were gavaged with the same volume water as the treatment group, while mice in treatment group were gavaged with water containing GA. Mice were maintained on the treatment for 16 weeks and were then sacrificed under isoflurane anesthesia after 16 hr fasting. Tissues were then rapidly removed, immediately frozen in liquid nitrogen, and stored at –80°C until needed for the metabolomics analysis. Other tissues were sampled and fixed in 10% neutral buffered formaldehyde for histological analysis. Serum samples were collected before the animals were sacrificed. Urine samples were collected before sacrifice and between 18∶00 p.m. and 00∶00 a.m. These samples were then snap-frozen in liquid nitrogen and stored at –80°C. Some urine samples were suspected to be contaminated based on some spurious fecal sample signals observed in their ^1^H NMR spectra. Therefore, these samples were excluded from the multivariate analysis.

The animals used in this study were housed and cared for in accordance with the NIH Guide for the care and use of laboratory animals. The experimental protocol was approved by the Animal Research Committee of National Defense Medical Center (IACUC-11-051).

### Serum Biochemistry Analysis

The activity levels of aspartate aminotransferase (AST) and alanine aminotransferase (ALT), and the levels of high density lipoprotein-cholesterol (HDL), triglycerides (TG), and total cholesterol (TCHO) were determined using an automatic blood chemistry analyzer Dry-Chem 4000i (Fujifilm, Saitama, Japan). Hemolysis was found to have occurred in some blood samples, which is known to interfere the AST and ALT measurement using blood samples. As a result the number of mice in the GA treatment group for AST and ALT analysis is nine. During insulin analysis, two serum samples were found to be too small to be analyzed. As a result number of animals in GAH and GAL groups is eight. All procedures completely complied with the manufacturer’s guidelines. Blood glucose concentrations were determined by a blood glucose meter (Accu-Check Advantage, Roche). Serum insulin levels were measured by ELISA kit (Linco Research, St. Charles, MO).

### Histological Analysis of Liver

For histopathological examination, the liver tissue samples were fixed in 10% neutral buffered formaldehyde, embedded in paraffin, and sectioned (4 µm). The sections were stained with hematoxylin and eosin.

### Sample Preparation and NMR Analysis of Serum, Urine and Tissue Samples

Sample preparation of the serum, urine and tissue samples for the metabolomics analysis were slightly modified from those previously described [32]. Serum and urine samples were thawed at room temperature, and then centrifuged at 13,000 rpm for 15 minutes to remove insoluble material. For serum preparation, 100 µl of serum was mixed with 500 µl of 0.9% NaCl (saline) in D_2_O. For urine preparation, 100 µl of urine was mixed with 300 µl D_2_O and 200 µl phosphate buffer (2.885 g Na_2_HPO_4_ and 0.525 g NaH_2_PO_4_ in 100 ml D_2_O, 1 mM TSP). Finally, 550 µl of each sample supernatants was placed in a 5 mm NMR tube for NMR analysis. Liver tissue samples (about 50 mg) were extracted with 0.685 mL of precooled methanol−water mixture (4/2.85, v/v) using a tissue lyser. After adding 0.4 ml chloroform to the methanol−water mixture, the solutions were separated into an upper methanol/water phase (with polar metabolites) and a lower chloroform phase (with lipophilic compounds). The chloroform phase solution was collected after centrifugation (1000×g, 4°C, 10 min) and chloroform was then removed *in vacuo*. The lipophilic extract was reconstituted using 600 µL of chloroform-*d* containing TMS. Then 550 µl of each sample was transfered to a 5 mm NMR tube for NMR analysis.

Analysis of the samples was performed as described previously [32] on an AVANCE AV-600 MHz spectrometer with a cryogenic probe. The serum was analyzed using the Carr-Purcell-Meiboom-Gill (CPMG) pulse sequence together with the one-dimensional nuclear overhauser enhancement spectroscopy (NOESY)-presat sequence in order to detect low molecular weight metabolites and using the bipolar-pair longitudinal-eddy-current (BPP-LED) pulse sequence in order to detect high molecular weight metabolites. The 1D J-resolved projection spectra were also used to help identify metabolites. The urine and lipophilic tissue extract was analyzed using the 1D NOESY-presat sequence and the 1D J-resolved projection spectrum.

All experiments were performed at 300 K. Manual shimming was performed on each samples to reach full width at half maximum (FWHM) <10 Hz on water peak of serum sample (using normal one-pulse sequence (zg) and with a line broadening of 0.3 hz) or <2.5 Hz on TSP peak of urine sample (using normal one-pulse sequence with water saturation (zgpr) and with a line broadening of 0.3 hz).

The 90° pulse length (∼14.0 µs) was adjusted individually for each sample. The free induction decays (FIDs) were acquired using 32 K data points with a spectral width of 20 ppm, and were zero-filled to 65536 points. A relaxation delay of 2.0 s was used. The other parameters were: number of scans (NS) = 128 and number of dummy scans (DS) = 16 for the CPMG experiments; NS = 128 and DS = 4 for the NOESY experiments; NS = 64 and DS = 4 for the LED-BPP experiments; and NS = 16 and DS = 16 for the J-resolved experiments.

### Data Processing and Analysis of NMR Data

The NMR spectra were automatically phased and baseline corrected using MestReNova software (8.0.2 Mestrelab research S.L.). The FIDs were multiplied by an exponential line-broadening factor of 0.3 Hz before Fourier transformation (FT). All spectra were referenced to the CH_3_ resonance of lactate at δ 1.33 ppm for the spectra obtained from plasma and to TSP at δ 0.00 ppm the spectra obtained from urine. Selected metabolite peaks were identified by comparing the results with the published literature (serum [33]–[36] and urine [33], [37]–[39]) and using the Chenomx NMR software suite (Version 7.5, Chenomx, Inc.).

For serum samples, each spectrum range of δ 0.04–10.0 was divided into integrated regions of equal width (0.005 ppm), whereas the range of δ 0.05–10.0 for urine samples was bucketed into 1990 bins (0.005 ppm). The regions containing resonance from residual water (δ 4.500–5.000) were excluded. When examining the urine samples, the urea peak is influenced by water presaturation and therefore the δ 5.000–6.000 region that contains the urea resonance was also excluded. The integral values of each spectrum of serum and urine samples were normalized to a total sum of all integrals in the spectrum in order to reduce any significant concentration differences between samples. For the tissue samples, the integral values of each spectrum were normalized against the weight of the wet tissue. The relative integrals of the liver cholesterol, liver triglyceride and liver fatty acids were calculated from the spectral regions at δ 0.670−0.695 for liver cholesterol (C18-H_3_), at δ 4.120−4.170 for liver triglyceride (Glycerol (C1-H^u^) and (C3-H^u^)) and at δ 0.81−0.93 for the methyl groups of all fatty acids (−CH_3_). The polyunsaturated fatty acids (PUFA)-to- monounsaturated fatty acids (MUFA) ratio was calculated from the spectral regions at δ 5.29−5.44 for unsaturated fatty acids (UFA) (−C***H*** = C***H***−), at δ 2.73−2.88 for PUFA (−C = C−C***H_2_***−C = C−) and at δ 0.81−0.93> for the methyl groups of all fatty acids (−CH_3_) [33]. The resulting datasets were then imported into SIMCA-P version 13.0 (Umetrics, Umea, Sweden), and all variables were scaled to Pareto (par) for the multivariate statistical analysis (principal components analysis (PCA), partial least squares discriminant analysis (PLS-DA), and orthogonal partial least squares discriminant analysis (OPLS-DA)). The quality of the fitting model can be explained by the appropriate R^2^ and Q^2^ values. R^2^ is defined as the total amount variation explained by the model and Q^2^ is the indicated predictability of the model under cross validation [33], [40]. As a result of the fact that ten mice were used to form the three different groups in this study, a cutoff value of |r| >0.576 (r >0.576 and r<- 0.576) was chosen for the correlation coefficient to be significant based on a discrimination significance of *p*<0.05. MCA were performed based on the followed criteria: 1. coefficient value |r| >0.576, 2. VIP>1, 3. *p* value <0.05.

### Statistical Analysis

All the results are shown as mean ± SE. Statistical analysis was carried out using one-way ANOVA followed by Bonferroni *post hoc* test. The criterion used for statistical significance was *p*<0.05.

## Results and Discussion

As a common natural product, GA is a naturally abundant plant phenolic compound in vegetables and fruits [3]–[6]. However, the effects and exact mechanisms by which GA affects NAFLD have not been totally described. Many mechanistic studies of GA have been performed using pharmacological methods [8], [10], [11], but these may not have reflected the effects of GA on metabolite profiles of the test organisms. In this study, we are the first to investigate the beneficial effects of GA administration on nutritional hepatosteatosis via a more holistic approach that uses NMR-based metabolomics.

### GA Ameliorates Hepatic Steatosis in HFD-induced NAFLD Mice

#### GA decreased body weight in HFD-induced NAFLD mice

In order to evaluate the preventive effects of GA on NAFLD, male C57BL/6 mice were subjected to HFD for 16 weeks. Previous studies have been indicated that hepatic steatosis is commonly associated with obesity [41]. Therefore, we measured body weight changes twice per week from the start point of the experiments to the end point of experiments. In this study we found that long-term HFD feeding resulted in a progressive increase in the body weight (**Figure S2A**) of the HFD-fed mice. Consistent with a previous study [10], we found that, compared with the HFD group mice, the mice treated with GA showed a reduced HFD-induced body weight gain (**Figure S2A**). In addition, food intake was not affected by GA treatment (**Figure S2B**), which indicates that the decrease of body weight found to occur with the GA treated mice were not due to changes in food consumption.

#### GA altered lipid homeostasis in HFD-induced NAFLD mice

**Figure 1:**
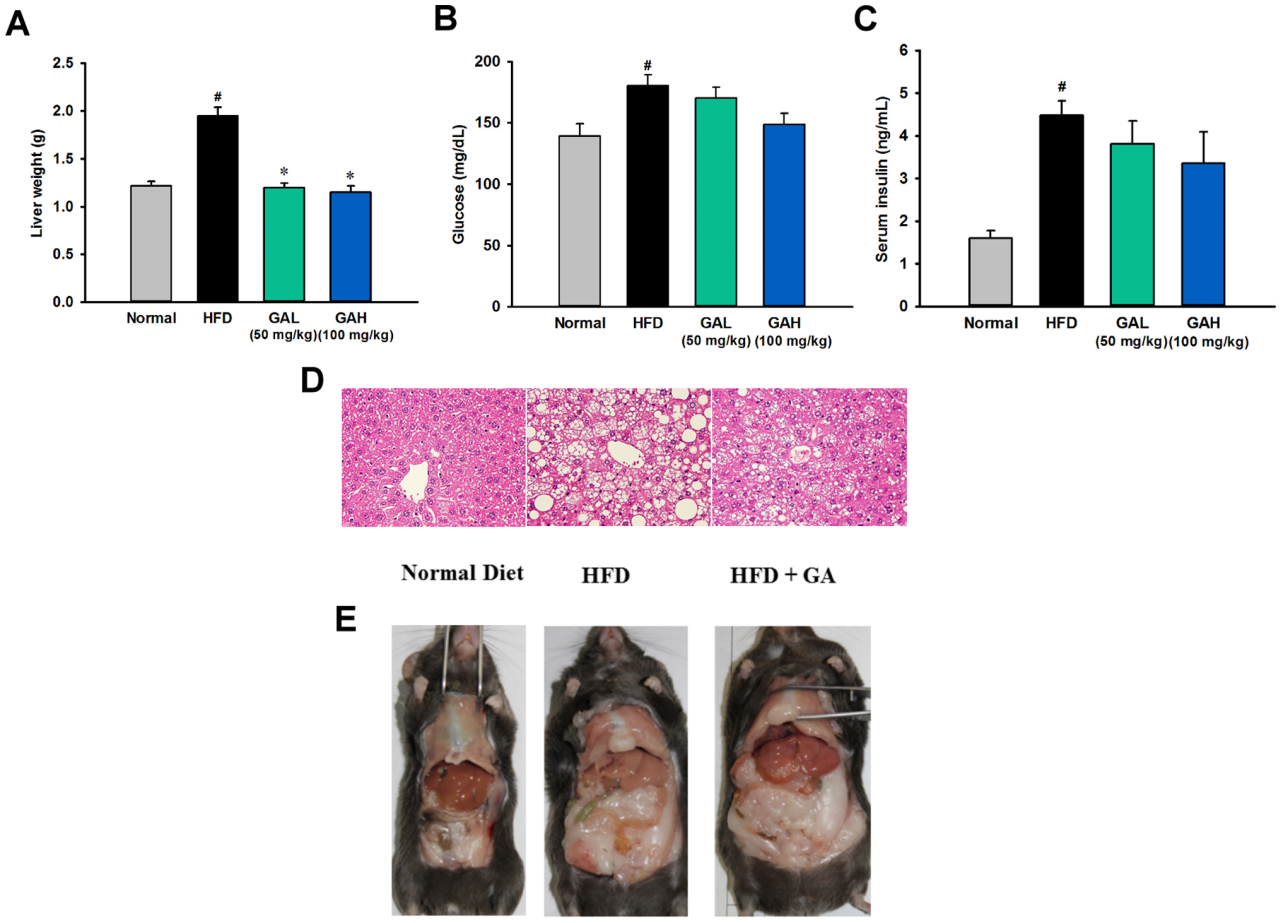
Gallic acid (GA) protects against hepatic steatosis and insulin resistance in high-fat diet-fed mice. (A) Effect of a high fat diet and gallic acid treatment on liver weight. (B–C) Administration of gallic acid for 16 weeks effectively improves glucose and insulin concentrations in mice fed the high fat diet. Serum insulin levels and blood glucose were assessed in mice fed a normal chow diet (normal group, n = 10), a high fat diet (HFD group, n = 11), and a high fat diet supplemented with GA (treatment group, high fat diet+GA, 50 (the number of mice used for the serum insulin analysis, n = 8; other experiments, n = 10) and 100 (the number of mice used for the serum insulin analysis, n = 8; other experiments, n = 10) mg/kg/day, orally). The data are presented as the mean ± SEM. #*p*<0.05, versus normal diet mice; *p<0.05, versus high fat diet-fed mice. (D and E) The gross morphology of the mouse livers and H&E staining of liver sections in different groups.

**Figure 2:**
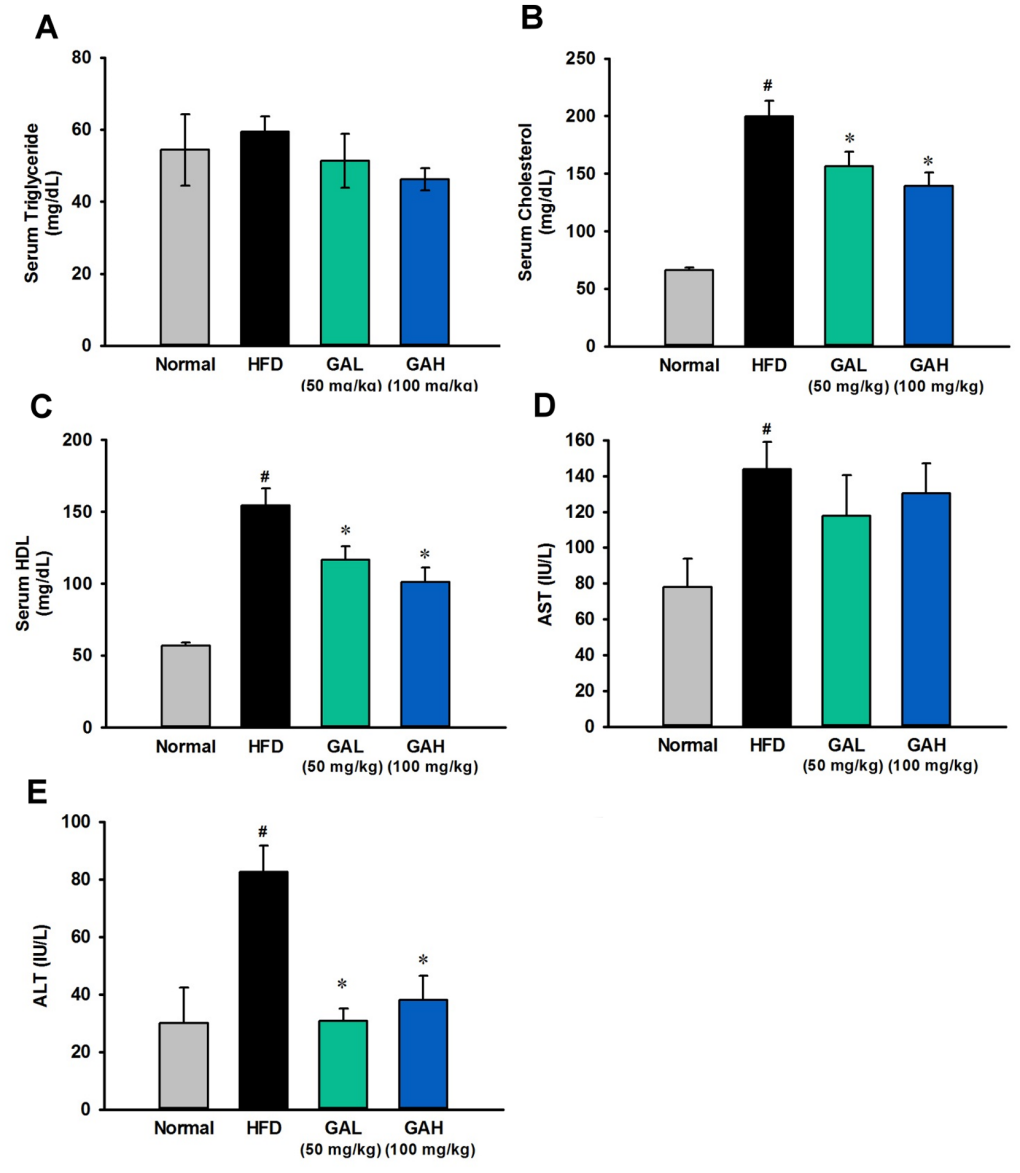
Gallic acid (GA) ameliorates changes in the serum biochemical parameters of mice with hepatic steatosis induced by high fat-diet feeding. (A) Serum triglyceride. (B) Serum cholesterol. (C) Serum high-density lipoprotein (HDL). (D) Aspartate aminotransferase (AST). (E) Alanine aminotransferase (ALT). The serum biochemical parameters were assessed in mice fed a normal chow diet (normal group, n = 10), a high fat diet (HFD group, n = 11), and a high fat diet supplemented with GA (treatment group, high fat diet+GA, 50 (the number of mice used for the AST and ALT analysis, n = 9; other experiments, n = 10) and 100 (the number of mice used for the AST and ALT analysis, n = 9; other experiments, n = 10) mg/kg/day). The data are presented as the mean ± SEM. #p<0.05, versus normal diet mice; *p<0.05, versus high fat diet-fed mice.

To evaluate whole-body glucose and lipid homeostasis, we next examined various systemic parameters in the mice. As expected, the HFD group mice have higher serum levels of HDL, TCHO, insulin and glucose than the normal diet group (**Figure 1A, 1B** **,** **Figure 2B, and 2C**). Interestingly, the serum TG level was not significantly affected by HFD feeding (**Figure 2A**). These clinical biochemistry results indicated that long-term HFD feeding caused severe insulin resistance (IR) and hypercholesterolemia, but did not induce hyperlipidemia. Compared with the HFD group mice, the GA treated mice showed a significant decrease in these serum metabolic parameters (**Figure 1A** **, and** **Figure 2B, 2C and 2E**). Although there are no statistically significant differences in blood glucose between the GA treatment group and the HFD group mice, the data showed that GA-treated mice have a recovering trend compared to those in the HFD group mice. The GA-treated mice developed only modest hypercholesterolemia, which demonstrates that GA treatment produced an improved lipid homeostasis found in the NAFLD mice.

#### GA reduced hepatic steatosis in HFD-induced NAFLD mice

**Figure 3:**
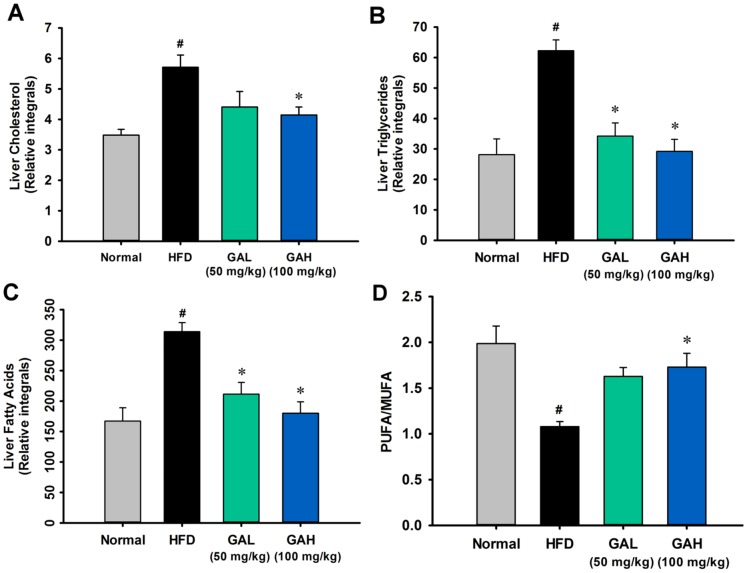
GA Gallic acid (GA) reduced liver lipid accumulation in high-fat diet-fed mice. (A) Liver cholesterol; (B) Liver triglyceride; (C) Liver fatty acids; (D) PUFA/MUFA ratio. The relative integrals of the liver cholesterol, liver triglyceride and liver fatty acids were calculated from the spectral regions at δ 0.670−0.695 for liver cholesterol (C18-H_3_), at δ 4.120−4.170 for liver triglyceride (Glycerol (C1-H^u^) and (C3-H^u^)) and at δ 0.81−0.93 for methyl groups of all fatty acids (−CH_3_). The PUFA-to-MUFA ratio was calculated from the spectral regions at δ 5.29−5.44 for UFA (−C***H*** = C***H***−), at δ 2.73−2.88 for PUFA (−C = C−C***H_2_***−C = C−) and at δ 0.81−0.93 for methyl groups of all fatty acids (−CH_3_) [33]. The data are presented as the mean ± SEM. #p<0.05, versus normal diet mice; *p<0.05, versus high fat diet-fed mice. The relative integrals were normalized against the weight of the wet tissue used for liver the extract.

HFD group mice exhibited increased liver weight (**Figure 1C**) and severe hepatosteatosis by both gross morphological examination and histological examination with the latter showing the liver as having hepatic vacuoles, lipid droplets and hepatocyte swelling (**Figure 1D, 1E**). Liver injury was also confirmed by significant increase of serum AST and ALT (**Figure 2D** **,** **Figure 2E**). In addition, HFD feeding caused a significantly increased level of liver TG, cholesterol and fatty acids and a significantly decreased ratio of PUFA to MUFA (**Figure 3**, **Table S4**). These findings indicated that HFD feeding resulted in significant hepatic steatosis and liver injury in mice. Based on the results of the NAFLD diagnostic gold standard, namely the histological analysis, these findings showed that the GA treatment had a significant hepatoprotective effect on HFD-induced steatosis (**Figure 1D**). Administration of GA reversed the excess fat accumulation in hepatic intracellular vacuole (**Figure 1D**), and reversed the increased level of liver TG, cholesterol and fatty acids. The significant decreased PUFA-to-MUFA ratio in HFD group was also reversed by GA treatment. GA treatment also protected liver function and lowered the increase in ALT level found in the HFD-fed mice (**Figure 2E**). Taken together, above results indicate that GA ameliorates hepatic steatosis in HFD-induced NAFLD mice.

In the present study, the treatment doses of GA are 100 mg/kg and 50 mg/kg, which are under the NOAEL of GA [42]. Based on formula from the FDA guidelines [43] that is used to convert an animal dose of 100 mg/kg and 50 mg/kg of GA to a human equivalent dose (HED), we calculated that the HED of GA are 487.8 mg/60 kg and 243.9 mg/60 kg, respectively.

### Metabolomics Profiling in Serum and Urine by ^1^H NMR Spectroscopy

**Figure 4:**
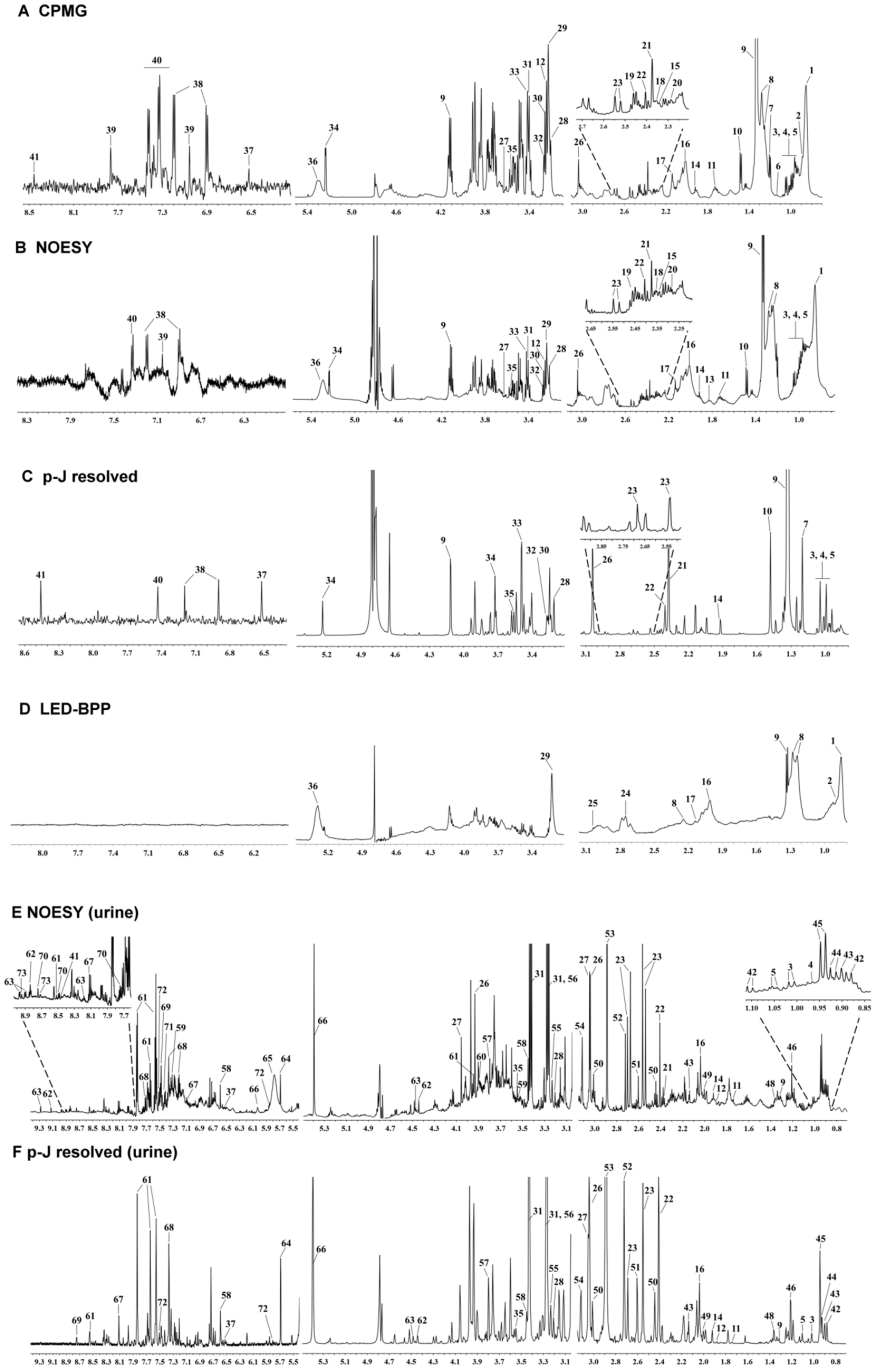
Typical 600 MHz ^1^H CPMG (A), NOESY (B), projected *J*-resolved (C),and BPP-LED (D) spectra of serum samples and ^1^H NOESY (E) and projected *J*-resolved (F) spectra of urine samples collected from the mice fed a normal chow diet at the 16 weeks. The keys metabolites in the serum and urine were assigned. The chemical shifts and peak multiplicity are described in **Table S1 and Table S2**.

To investigate the biochemical effects of HFD-induced hepatosteatosis and of GA intervention in NAFLD mice, we performed an ^1^H NMR-based metabolomics analysis combined with pattern recognition techniques to detect the endogenous metabolites present in the serum and urine of the control, HFD and treatment group mice (**Figure S1B**). Typical 1D ^1^H NMR spectra of the serum and urine taken from normal group mice are presented in **Figure 4**. A total of 73 endogenous metabolites were unambiguously assigned based on the published literature [33]–[39] and these were confirmed by Chenomx 7.6.

In this study, serum metabolic profiling provides information on lipid and energy metabolism (**Table S2**). When a typical 600-MHS ^1^H NMR BPP-LED spectrum is analyzed, serum signals characterizing common markers of CH_3_ resonance come from components of lipoprotein, such as cholesterol, HDL, LDL, and phospholipids. In addition, the serum CPMG and NOESY spectra contained resonances signals from low-molecular-mass metabolites, such as branched-chain amino acids (BCAAs: valine, isoleucine, leucine), acidic amino acids (glutamate, glycine), basic amino acids (lysine, arginine), aromatic amino acids (tyrosine, phenylalanine), other aliphatic amino acids (alanine, proline), 1-methylhistidine, ketone bodies (3-hydroxybutyrate and acetoacetate), several carboxylic acids (acetate, formate), various choline-associated metabolites (choline, trimethylamine N-oxide (TMAO), betaine) and taurine. A number of glycolysis and tricarboxylic acid-cycle (TCA cycle) related metabolites and intermediates (glucose, pyruvate, lactate, succinate, fumarate, citrate) were also detected in the serum.

The urine metabolic profiling provides information on intermediary metabolism (**Table S3**). A typical urine ^1^H NMR spectrum was found to show a range of different metabolites including amino acid (isoleucine, leucine, valine, lysine, and arginine, glycine), organic acids (formate, acetate, butyrate), TCA cycle metabolites (succinate, citrate, fumarate), gut microbiota-derived metabolites (methylamine, dimethylamine (DMA), trimethylamine (TMA), TMAO, hippurate, formate, benzoate), nicotinate and nicotinamide metabolism derived metabolites (trigonelline, 1-methylnicotinamide, nicotinamide N-oxide, niacinamide), choline, creatine, creatinine, and urea.

### Evaluation of the HFD-induced Hepatosteatosis Model Using NMR-base Metabolomics Approach

In order to identify the various different metabolic changes affecting the NAFLD mice, the NMR spectrum were preprocessed in order to be able to carry out multivariate statistical analysis (PCA, PLS-DA, and OPLS-DA). First, an unsupervised pattern recognition method, PCA, was performed. Exploratory PCA was employed to detect intrinsic clustering and possible outliers [44]. The different PCA score plots of the serum illustrates that the HFD group is clearly separated from the normal group (**Figure S3A–C,** (A) CPMG: R^2^X = 0.589, Q^2^ = 0.448; (B) NOESY: R^2^X = 0.667, Q^2^ = 0.539; (C)BPP-LED: R^2^X = 0.742, Q^2^ = 0.475) or urine (**Figure S3D,** NOESY: R^2^X = 0.71, Q^2^ = 0.583**)**. The major effect of component T1 is to differentiate the HFD induction on the PCA score plot. Hotelling’s T2 statistical results indicated that only one outlier observation was found within the PCA score plot of the BPP-LED spectrum. These findings demonstrated that the preprocessed dataset from the NMR spectrum has good stability and low variation.

For the regression analysis, a supervised pattern recognition method, PLS-DA, was used. The PLS-DA model was validated using a 7-fold cross validation model and then was further evaluated using a permutation test (200 permutations). The quality of the model was assessed by the cross-validation parameter (Q^2^Y), which indicates the predictability of the model [45]. By applying PLS-DA, a reasonably good separation was obtained for the scatter plots obtained from the serum (**Figure S4A**, CPMG: R^2^X = 0.41, R^2^Y = 0.932, Q^2^ = 0.63; **Figure S4C**, NOESY: R^2^X = 0.657, R^2^Y = 0.841, Q^2^ = 0.753; **Figure S4E**, BPP-LED: R^2^X = 0.711, R^2^Y = 0.929, Q^2^ = 0.873) and urine (**Figure S4G**, NOESY: R^2^X = 0.705, R^2^Y = 0.988, Q^2^ = 0.973) samples. In a similar manner to that of the PCA score plot, the major effect of principal component T1 discriminates diet induction for the serum and urine samples. To further validate the PLS-DA model, permutation tests were performed (**Figure S4**, **right part**). A higher Q^2^ was obtained from the real model than was obtained as a Q^2^_max_ by the permutation test when the normal group was compared with the HFD group. Similarly, the R^2^ of the real model was higher than the R^2^_max_ obtained from the permutation test. These findings imply that the PLS-DA model possessed great predictability between the normal group and the HFD group and are not overfitted.

**Figure 5:**
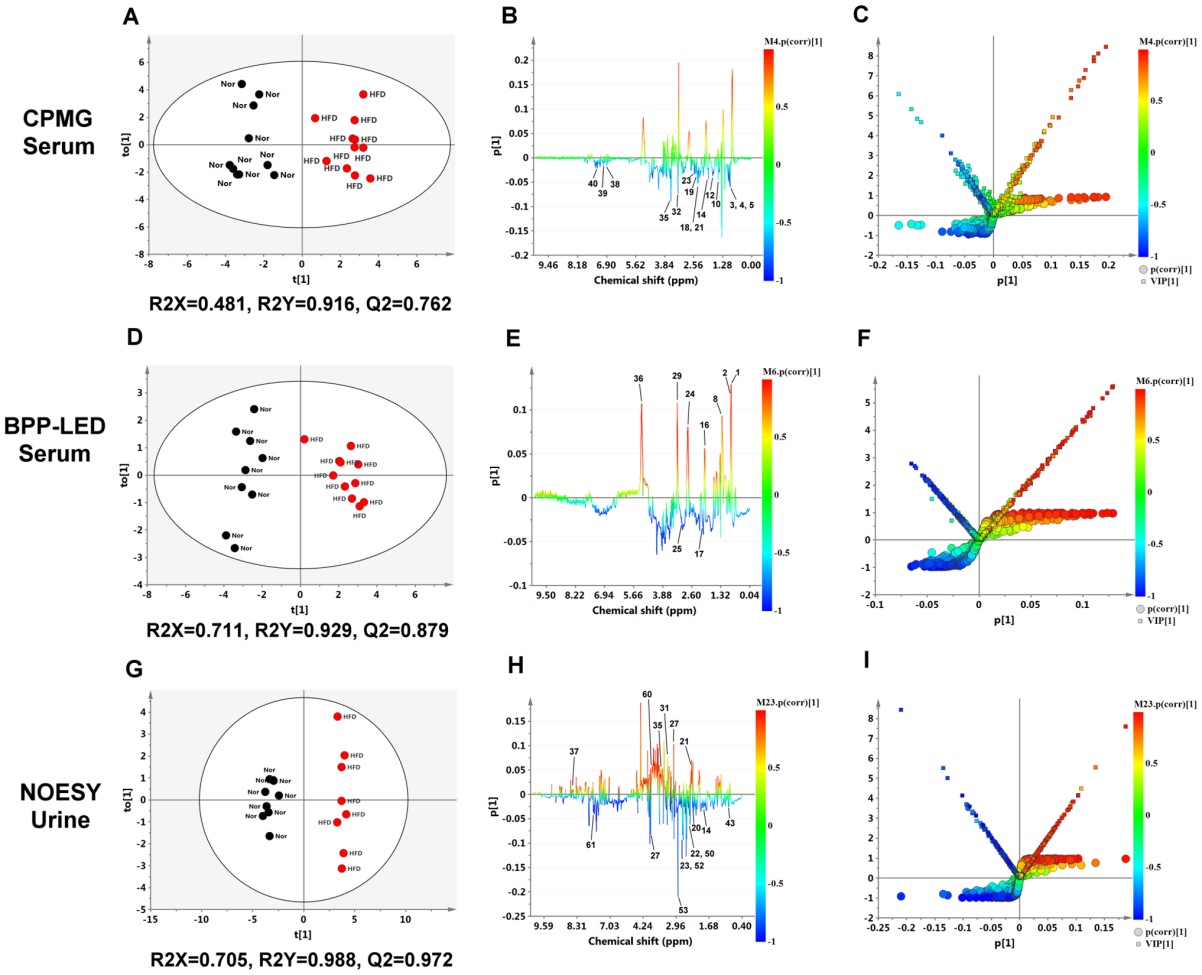
O-PLS-DA results for normal chow diet and high fat diet-fed mice derived from ^1^H NMR CPMG spectra of serum (A, B, C), BPP-LED spectra of serum (D, E, F), and NOESY spectra of urine (G, H, I). O-PLS-DA scores plots (A, D, G), coefficient-coded loadings plots (B, E, H), and the S-plot combined with the VIP plot and color coefficient scale bar (C, F, I) for the models discriminating the normal group (black filled dots) and HFD groups (red filled dots) based on data for plasma and urine. Metabolite keys to the number are shown in **Table 1** **and** **Table 2** **.** Nor, normal group; HFD, high fat diet group.

As a result of the above, the OPLS-DA method was employed in order to maximize the covariance between the measured data (peak intensities in NMR spectra) and the response variable (predictive classifications). The scores plot of the OPLS-DA show a much clearer separation between the normal group and the HFD group and have higher R^2^ and Q^2^ values than the other pattern recognition methods (**Figure 5A**, serum CPMG spectra: R^2^X = 0.481, R^2^Y = 0.916, Q^2^ = 0.762; **Figure 5D**, serum BPP-LED spectra: R^2^X = 0.711, R^2^Y = 0.929, Q^2^ = 0.879; **Figure 5G**, urine NOESY spectra: R^2^X = 0.705, R^2^Y = 0.988, Q^2^ = 0.972). These findings indicated that OPLS-DA should be able to help us to identify the important and latent variables associated with liver steatosis and as well as those related to the drug-intervention mechanism.

We further used OPLS-DA with coefficient plots to directly visualize the results of the loadings and correlation coefficients (**Figure 5B, 5E and 5H**). The color-coded correlation coefficients indicate the significance of the metabolites in terms of their contribution to the separation between the different groups [46], [47]. The metabolites that are colored red and blue are more significant than those that are colored green. Additionally, to exhibit how each of these variables is responsible for the separation more intuitively, we created an S-plot that is combined with a VIP plot and a color coefficient scale bar (we called this the “SV coefficient plot”) (**Figure 5C, 5F, 5I**). This is the first time that a “SV coefficient plot” has been used in ‘omics related papers. The SV coefficient plot is able to provide rich information, including the metabolite contribution of first components (PC1), the VIP values of the metabolites, and Pearson’s correlation coefficient values (*p*(corr)), all in one figure. Hence, the SV coefficient plot should be able to be applied generally to potential biomarker selection in MCA.

The two different plots, the coefficient-coded loadings plots (**Figure 5B, 5E, 5H**), and the SV coefficient plot (**Figure 5C, 5F, 5I**), show that various latent metabolites in the serum and urine have significant values. A series of key metabolites contribute to the separation of the HFD group from the normal group; these, along with their significance values (coefficient value, VIP value, and p value), are summarized in **Table 1** and **Table 2**. When the HFD group is compared with the normal group, the levels of the metabolites with a positive coefficient value were found to have been increased by HFD feeding, whereas those with negative values were found to have been decreased (**Table 1** **and** **Table 2**). These findings demonstrate that the selected metabolites that have higher or lower coefficient and VIP values are highly relevant biomarkers when explaining the discrimination between the different groups. Moreover, in order to verify the results of the OPLS-DA, the NMR spectra integrals of the altered metabolites were compared using independent Student’s *t*-testa (**Table 1** **,** **Table 2** **and** **Table 3**).

**Table 1:** OPLS-DA coefficients and their variable importance in projection (VIP) for significantly changed metabolites in serum.

Metabolites			Multi-criteria assessment (MCA)^c^					
No.	Names	δ ^1^H (ppm)	HFD *vs*. Normal			HFD+GAH *vs*. HFD		
			Coefficient value^d^	VIP	*p* value^e^	Coefficient value^d^	VIP	*p* value^e^
**Large macromolecules** a								
1	HDL	0.86 (m)	0.974	5.59	↑, <0.001	−0.777	5.18	↓, 0.001
2	LDL	0.88 (m)	0.953	4.06	↑, <0.001	−0.817	3.88	↓, 0.001
8	Lipid	1.28 (m)	0.857	4.00	↑, <0.001	−0.882	4.77	↓, <0.001
16	N-acetyl-glycoprotein	2.04 (s)	0.882	2.36	↑, <0.001	−0.840	2.50	↓, 0.001
17	O-acetyl-glycoprotein	2.14 (s)	−0.896	1.09	↓, <0.001	0.610	1.18	↑, 0.003
24	PUFA	2.75 (m)	0.985	2.93	↑, <0.001	−0.776	2.25	↓, 0.001
25	Albumin	3.02	−0.947	1.43	↓, <0.001	0.776	1.39	↑, 0.002
29	Phosphotidylcholine	3.23 (s)	0.904	4.52	↑, <0.001	−0.860	5.85	↓, <0.001
36	UFA	5.29 (m)	0.983	4.37	↑, <0.001	−0.838	3.93	↓, <0.001
**Small molecules** b								
3	Isoleucine	1.01 (d)	−0.915	1.56	↓, <0.001	0.744	1.14	↑, 0.031
4	Leucine	0.97 (t)	−0.732	2.07	↓, 0.001	0.713	1.83	↑, 0.001
5	Valine	1.04 (d)	−0.755	2.10	↓, <0.001	0.628	1.61	↑, 0.010
6	Isobutyrate	1.14 (d)	−0.681	0.92	↓, 0.003	0.605	1.15	↑, 0.008
7	3-hydroxybutyrate	1.20 (d)	−0.507	2.18	–, 0.113	0.584	5.24	↑, 0.017
9	Lactate	4.11 (q)	−0.683	2.10	–, 0.285	0.601	2.77	–, 0.124
10	Alanine	1.48 (d)	−0.585	1.93	↓, 0.013	0.519	1.76	–, 0.067
11	Lysine	3.03 (t)	−0.815	1.19	↓, 0.001	0.791	1.87	↑, 0.001
12	Arginine	1.67 (m)	−0.701	0.98	↓, 0.003	0.711	0.99	↑, 0.003
13	Ornithine	3.06 (t)	−0.303	0.68	–, 0.254	0.742	1.28	↑, 0.005
14	Acetate	1.92 (s)	−0.742	1.83	↓, 0.003	0.568	1.17	–, 0.078
18	Glutamate	2.35 (t)	−0.843	1.21	↓, 0.003	0.834	1.45	↑, 0.006
19	Glutamine	2.45 (m)	−0.614	1.30	↓, <0.001	0.737	1.83	↑, 0.001
20	Acetoacetate	2.29 (s)	−0.591	1.23	–, 0.051	0.664	2.50	↑, 0.005
21	Pyruvate	2.37 (s)	−0.818	1.48	↓, <0.001	0.646	1.47	↑, 0.031
23	Citrate	2.53 (d)	−0.786	1.31	↓, <0.001	0.492	0.71	–, 0.136
32	Betaine	3.27 (s)	−0.774	2.87	↓, 0.001	0.623	2.21	–, 0.128
35	Glycine	3.56 (s)	−0.722	2.93	↓, 0.001	0.737	3.34	↑, 0.004
38	Tyrosine	6.90 (d)	−0.775	0.70	↓, <0.001	0.811	0.92	↑, 0.002
39	1-Methylhistidine	7.06 (s)	−0.760	0.69	↓, 0.001	0.779	0.65	↑, 0.003
40	Phenylalanine	7.33 (m)	−0.920	0.65	↓, <0.001	0.837	0.62	↑, 0.003

a Coefficient values are calculated by LED-BPP coefficient-coded loadings plots.

b Coefficient values are calculated by CPMG coefficient-coded loadings plots.

c Multi-criteria assessment (MCA) was performed by followed criteria: 1. Coefficient value |r| >0.576, 2. VIP>1, 3. *p* value <0.05.

d The coefficients from the OPLS-DA results; positive and negative signs indicate positive and negative correlations in the concentrations of serum metabolites.

e Independent t test (two-tailed).

HDL: high-density lipoprotein; LDL: low-density lipoprotein; PUFA: Polyunsaturated fatty acids; UFA: unsaturated fatty acids.

**Table 2:** OPLS-DA coefficients and their variable importance in projection (VIP) for significantly changed metabolites in urine.

Metabolites			Multi-criteria assessment (MCA)^a^					
No.	Names	δ ^1^H (ppm)	HFD vs. Normal			HFD+GAH vs. HFD		
			Coefficient value^b^	VIP	*p* value^c^	Coefficient value^b^	VIP	*p* value^c^
14	Acetate	1.92 (s)	0.662	1.50	–, 0.080	0.594	1.30	↑, 0.044
20	Acetoacetate	2.29 (s)	−0.920	1.38	↓, <0.001	0.519	1.75	↑, 0.040
21	Pyruvate	2.37 (s)	0.863	1.98	↑, <0.001	0.020	0.34	–, 0.801
22	Succinate	2.41 (s)	−0.842	2.43	↓, <0.001	0.653	3.36	–, 0.081
23	Citrate	2.69 (d)	−0.701	3.70	↓, 0.005	0.681	2.48	–, 0.970
26	Creatine	3.93 (s)	−0.750	3.32	↓, 0.001	0.076	1.38	–, 0.564
27	Creatinine	3.05 (s)	0.938	4.14	↑, <0.001	0.641	3.31	↑, 0.048
31	Taurine	3.27 (t)	0.639	3.07	↑, 0.006	−0.653	7.55	↓, 0.019
35	Glycine	3.55 (s)	0.738	2.07	↑, 0.001	−0.312	1.61	–, 0.222
37	Formate	8.46 (s)	0.909	0.42	↑,<0.001	−0.396	0.32	–, 0.179
43	Butyrate	0.90 (t)	0.619	1.90	↑, 0.001	0.311	1.65	–, 0.063
50	2-oxoglutaric acid (2-KG)	2.44 (t)	−0.782	1.66	↓, 0.001	0.267	0.19	–, 0.787
52	Dimethylamine (DMA)	2.72 (s)	−0.412	0.80	–, 0.089	−0.472	1.89	↓, 0.041
53	Trimethylamine (TMA)	2.89 (s)	−0.915	8.43	↓, <0.001	−0.566	5.56	↓, 0.031
57	Guanidoacetate	3.80 (s)	0.951	2.74	↑, <0.001	−0.363	1.61	–, 0.236
58	Trans-aconitate	6.59 (s)	−0.872	1.13	↓, <0.001	0.203	0.12	–, 0.684
60	Glucose (α & β form)	3.90 (dd)	0.835	1.73	↑, <0.001	−0.588	2.40	↓, 0.013
61	Hippurate	7.64 (t)	−0.970	1.58	↓, <0.001	0.516	0.56	↑, 0.035
62	Trigonelline	8.84 (t)	−0.951	0.64	↓, <0.001	0.449	0.30	–, 0.129
63	1-Methylnicotinamide	8.90 (d)	0.975	0.67	↑, <0.001	−0.330	0.42	–, 0.137
70	Nicotinamide N-oxide	8.49 (d)	0.573	0.23	↑, <0.001	−0.549	0.54	–, 0.261
73	Niacinamide	8.94 (d)	−0.635	0.22	↓, 0.005	0.355	0.09	–, 0.543

a Multi-criteria assessment (MCA) was performed using the followed criteria: 1. Coefficient value |r| >0.576, 2. VIP>1, 3. *p* value <0.05.

b Coefficient values were calculated using NOESY coefficient-coded loadings plots. The coefficients from the OPLS-DA results; positive and negative signs indicate positive and negative correlations in the concentrations of serum metabolites.

c Independent t test (two-tailed).

**Table 3:** Normalized integral values of serum and urine metabolites from NMR spectra.

Metabolite	Normalized integral value (NMR signal×10^3^)^a^			Samples
	Normal	HFD^b^	GAH^b^	
**Glycolysis and TCA cycle related metabolites and intermediates**				
Pyruvate	62.86±3.73	41.44±2.06#	52.44±4.42*	Serum
Acetate	92.18±9.24	55.01±3.07#	63.45±3.35	
Glucose	219.48±4.48	276.05±9.15#	236.86±10.28*	Urine
Pyruvate	119.1±3.68	190.97±10.94#	184.03±24.54	
Acetate	126.22±1.81	121.25±1.94#	134.28±5.21*	
Lactate	606.89±390.01	163.85±9.28	190.94±14.44	Serum
Citrate	45.11±3.56	27.79±1.18#	31.37±2.04	
Citrate	363.73±82.03	47.15±6.31#	103.04±28.93	Urine
2-ketoglutarate	132.62±11.99	74.58±3.89#	76.54±5.98	
Formate	0.06±0.06	1.95±0.24#	1.37±0.55	
Succinate	227.09±20.09	112.49±3.09#	216.11±55.05	
Trans-aconitate	23.52±3.29	0.41±0.30#	0.79±1.19	
**Urea cycle**				
Arginine	37.92±3.49	26.24±0.80#	30.08±0.78*	Serum
Ornithine	43.2±11.9	29.78±1.46	36.39±1.45*	
**Lipid metabolism**				
HDL	460.19±19.83	728.02±18.37#	626.13±19.23*	Serum
LDL	290.11±5.92	426.34±10.84#	368.21±11.16*	
Lipid	520.17±23.79	681.25±14.05#	600.46±11.86*	
PUFA	119.39±5.31	191±3.5#	171.87±3.48*	
UFA	187.48±12.27	348.32±8.69#	293.82±8.79*	
**Plasma protein**				
Albumin	76.41±1.26	60.2±1.5#	67.83±1.46*	Serum
N-acetyl-glycoprotein	196.49±4.2	246.5±4.57#	222.8±3.92*	
O-acetyl-glycoprotein	105.46±1.59	96.7±0.97#	102.4±1.45*	
**Ketogenesis**				
Acetoacetate	68.16±10.45	44.48±1.71	68.02±6.49*	Serum
3-hydroxybutyrate	241.1±52.06	149.01±9.17	266.37±40.07*	
Acetoacetate	132.23±1.81	100.12±2.8#	123.24±9.08*	Urine
**BCAA metabolism and amino acids metabolism**				
Isoleucine	57.13±5.76	23.34±2.18#	32.29±3.25*	Serum
Leucine	144.56±12.32	97.27±1.94#	109.28±2.36*	
Valine	102.71±10.55	56.78±2.20#	68.26±3.46*	
Glutamate	46.13±2.42	32.04±1.24#	39.5±1.39*	
Glutamine	47.4±5.84	26.61±2.58#	40.61±3.88*	
Tyrosine	8.36±0.87	3.6±0.32#	6.78±0.85*	
Phenylalanine	8.59±0.47	4.92±0.26#	6.44±0.39*	
Alanine	127±15.52	77.65±7.19#	96.11±6.07	
Lysine	77.58±2.83	62.16±2.45#	74.93±2.24*	
**Bile acid metabolism**				
Glycine	194.75±23.23	101.43±4.57#	142.91±10.6*	Serum
Glycine	145.23±5.28	239.26±24.18#	206.51±8.45	Urine
Taurine	648.94±40.34	881.24±61.44#	489.81±125.78*	
**Creatine metabolism**				
Guanidoacetate	269.45±6.09	393.24±8.11#	360.04±24.75	Urine
Creatine	444.41±49.18	208.92±18.72#	258.75±82.26	
Creatinine	511.55±23.05	802.37±18.7#	890.01±34.46*	
**Methylamine metabolism**				
Betaine	257.76±13.90	167.77±16.58#	202.12±13.41	Serum
Phosphotidylcholine	450.87±18.55	625.44±18.94#	506.70±17.40*	
**Gut microbiota-related metabolism**				
Isobutyrate	26.41±2.26	17.16±0.73#	22.49±1.55*	Serum
Butyrate	187.32±3.36	163.46±5.24#	187.08±10.46*	Urine
Hippurate	48.22±2.32	7.87±0.55#	10.33±0.9*	
Dimethylamine (DMA)	250.44±9.68	226.46±8.75	197.9±9.2*	
Trimethylamine (TMA)	1737.64±125.07	504.72±61.46#	269.21±76.77*	
**Histidine metabolism**				
1-methylhistidine	5.74±0.90	1.09±0.17#	2.74±0.48*	Serum
**Nicotinate and nicotinamide metabolism**				
Nicotinamide (niacinamide)	1.32±0.31	0.16±0.16#	0.36±0.30	Urine
1-Methylnicotinamide	0.33±0.15	7.69±0.44#	5.9±1.01	
Trigonelline	5.64±0.55	0.00±0.00#	0.44±0.22	

a The data are presented as the mean ± SEM.

b Independent t test (two-tailed).

# *p*<0.05, versus normal diet mice;

*p<0.05, versus high fat diet-fed mice.

TCA cycle: tricarboxylic acid-cycle.

The observed latent metabolites identified as being associated with lipid metabolism (HDL, LDL, TG, fatty acids, polyunsaturated fatty acids, unsaturated fatty acids), ketogenesis (acetoacetate, 3-hydroxybutyrate), protein metabolism marker (albumin), liver injury biomarker (albumin, taurine), glycolysis (lactate, pyruvate) and TCA cycle intermediates (citrate, succinate, and 2-ketoglutarate), amino acids metabolism (BCAAs, aromatic, acidic, basic, and other aliphatic amino acid), choline metabolism (Phosphotidylcholine, betaine) and gut-microbiota metabolism (TMA, DMA, hippurate, butyrate, isobutyrate), nicotinate and nicotinamide metabolism (trigonelline, 1-methylnicotinamide, nicotinamide N-oxide, niacinamide), and creatine metabolism (creatine, creatinine, guanidoacetate) (**Figure 7** **and** **Table 3**).

### Evaluation of the GA Therapeutic Effect on the HFD-induced Hepatosteatosis Mice using a Supervised Pattern Recognition Method, PLS-DA

**Figure 6:**
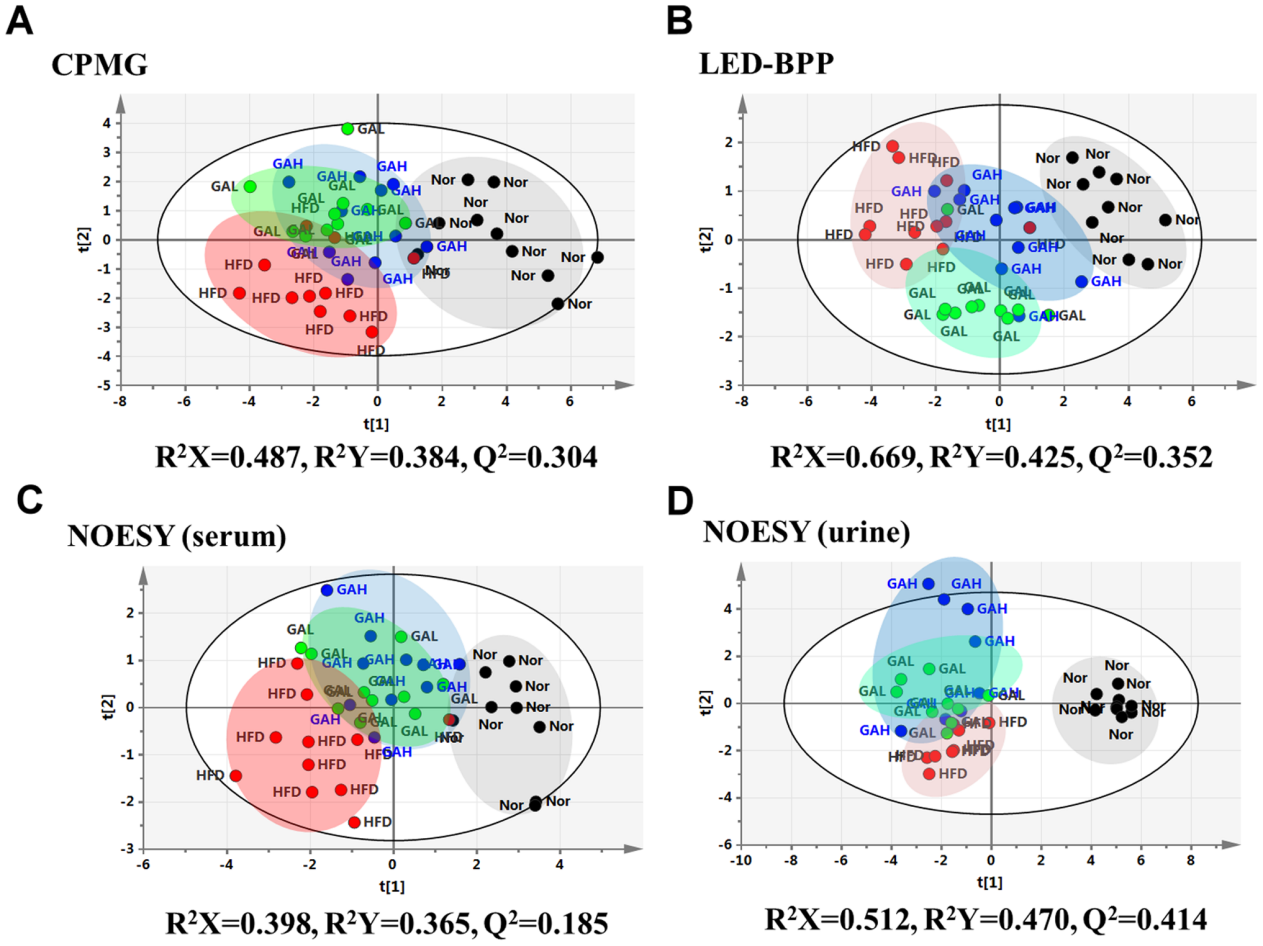
PLS-DA scores plots for (A) standard 1D CPMG spectra of serum, (B) NOESY spectra of serum, (C) BPP-LED spectra of serum, and (D) NOESY spectra of urine from normal group, high fat diet group, and GA treatment group. Nor, normal group; HFD, high fat diet group; GAH and GAL, high and low dose of GA treatment group.

The serum PLS-DA model is able to discriminate the effect of HFD induction and GA intervention on the score plot (**Figure 6A–C**). Using the CPMG score plot (**Figure 6A**), the HFD group is clearly separated from the normal group in the direction of component T1, which implies that the metabolic characteristics of the various small molecules are distinctively different. However, a few GA-treated mice are close to HFD-fed mice, while other GA-treated mice are close to the normal group mice. These differences suggest that GA treatment is not able to restore the homeostasis of all the disturbed metabolic pathways of the HFD-fed mice to the state found in the normal group mice. Using the NOESY and LED-BPP score plots (**Figure 6B, 6C**), it can be seen that the GA-treated group is located in a distinct cluster that is different to those of the HFD group and normal group; furthermore, the GA-treated group is closer to the normal group than the HFD group. This supports the hypothesis that GA treatment affects the NAFLD mice by improving the homeostasis of the mice’s serum at a macromolecular level.

**Figure 7:**
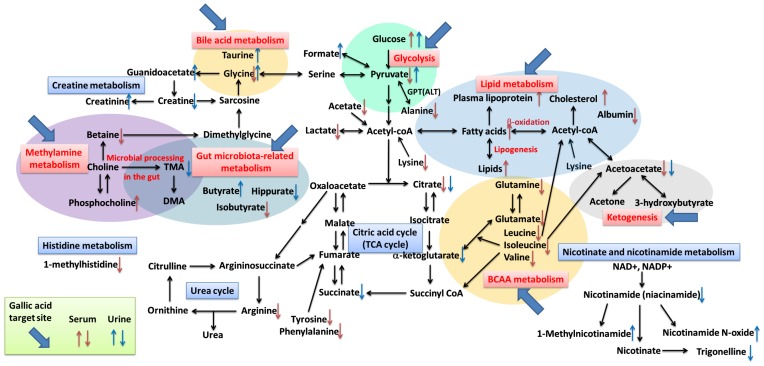
Disturbed metabolic pathways in high-fat diet-induced hepatosteatosis mice. The metabolic pathways where it was that gallic acid treatment was able to intervene are indicated by blue arrows. ↑, Up-regulated; ↓, down-regulated; red color, serum; blue color, urine. BCAA, Branched-chain amino acids; TCA cycle: tricarboxylic acid-cycle; TMA: Trimethylamine; DMA: Dimethylamine.

The urine PLS-DA model also clearly differentiates the three different groups (normal group, HFD group, and treatment group) based on the score plot result (**Figure 6D**). The main effect of component T1 is to discriminate HFD induction, whereas T2 seems to describe the response of GA intervention to HFD induction. Taken together, the results of the metabolomic analysis of the metabolic effects of GA support those obtained by biochemistry and histopathology and confirm the hypothesis that GA has a significant therapeutic effect on NAFLD mice.

### Metabolic Effects of GA in High Fat Diet-induced NAFLD Mice: Traditional Biochemical Aspect

#### Lipid metabolism and ketogenesis

HFD feeding resulted in significant dyslipidemia, including elevated levels of lipoprotein and fatty acids (**Table 1** **,** **Figure 3**). Compared with the normal group, there were significantly increased levels of phosphotidylcholine and O-acetyl-glycoprotein in the serum of the HFD-fed mice, which is consistent with the swelling of hepatocytes (**Figure 1D**). Phophocholine is an abundant structural component of the cell membrane [47], [48]. Serum O-acetyl-glycoprotein is an “acute phase” glycoprotein that is associated with inflammation of injury tissue in inflammatory animal models [47], [49]. Previous studies have suggested that elevated O-acetyl glycoprotein fragment signals in the blood are associated with inflammatory associated diseases, including cancer, certain liver diseases, and also surgical trauma [49], [50]. GA was able to reduce these increased levels of metabolites, which indicates that GA ameliorates hepatosteatosis and protects the liver against injury during HFD-feeding (**Figure 1D** **and** **Figure 2E**).

Ketone bodies, which contain acetone, acetoacetate and 3-hydroxybutyrate, are important by-products of β-oxidation of fatty acids in the human body [51]. They are produced from acetyl-CoA by ketogenesis and this mainly occurs in the mitochondrial matrix of hepatocytes. Previous studies have been revealed that long term HFD feeding causes hepatocyte mitochondrial DNA damage and dysfunction, and that, as a result, there is increased oxidative stress in the liver [52]. In the present study, the levels of acetoacetate and 3-hydroxybutyrate were lower in the HFD-fed mice than in the normal diet-fed mice (**Table 1** **,** **Table 2** **and** **Table 3** **),** which suggests that HFD feeding caused a certain degree of mitochondria dysfunction in the mouse hepatocytes, thereby decreasing the β-oxidation of fatty acids in the liver. It is worth noting that GA treatment increased the levels of ketone bodies in the serum and urine (**Table 1** **,** **Table 2** **and** **Table 3**). In addition, an elevated concentration of acetate, which is the end product of fatty acid oxidation in peroxisomes [53], was also found in the GA-treated mice (**Table 1** **and** **Table 3**). These results demonstrate that the liver protective effect of GA is partially due to an increase β-oxidation of fatty acids in the liver. Previous studies have suggested that a decreased PUFA/MUFA ratio is indicative of excessive lipid peroxidation and oxidative stress in HFD-fed mice [54]. The PUFA/MUFA ratios in the liver were calculated (Figure 3D). HFD feeding caused a significant decreased the ratio of PUFA to MUFA (Figure 3D) and GA reversed this phenomenon. Our findings are consistent with previous studies [54].

#### Albumin

Long-term HFD feeding caused a significant decrease in the level of serum albumin in HFD-fed mice (**Table 1** **and** **Table 3**). Previous studies have demonstrated that insulin dysfunction is caused by HFD feeding and that this inevitably results in increased protein catabolism. This increase produces precursors for gluconeogenesis and energy generation via the TCA cycle, and decreased protein production [55]. On the other hand, albumin is a protein produced specifically by the liver. Therefore, the serum level of albumin ought to reflect liver function [56]. In general, serum albumin levels are decreased when chronic liver disease, such as hepatitis or liver cirrhosis, is present. However, the relationship between serum albumin level and hepatic steatosis is unclear. We suggested that the decreased level of serum albumin is probably a symptom of reduced liver function in the hepatosteatosis mice (**Table 1** **and** **Table 3**). It is worth noting that GA treatment is able to restore to some degree the reduced level of serum albumin in HFD fed mice. The relationship between albumin and NAFLD diagnosis remains unclear and should be investigated in the future.

#### Glycolysis and TCA cycle (energy metabolism)

HFD feeding induced a significant decrease in the levels of both anaerobic (lactate) and aerobic glycolysis metabolites (pyruvate) (**Table 1** **and** **Table 3**) as well as increased levels of serum glucose and insulin (**Figure 1B and 1C**). These disorders of glucose metabolism indicated that the occurrence of enhanced gluconeogenesis and decreased glycolysis in the HFD group mice. This is consistent with the fact that lipid accumulation in the liver impairs insulin signaling and the ability of insulin to regulate gluconeogenesis [57]. In addition to abnormal glucose metabolism, a disordered energy metabolism is the other main biological phenotype associated with long term HFD feeding. Lipid accumulation-induced mitochondria DNA damage correlates with mitochondrial dysfunction and increased oxidative stress in skeletal muscle and liver, which are associated with the induction of endoplasmic reticulum stress markers ER stress, protein degradation and apoptosis [52]. In present study, compared with the normal group mice, various TCA cycle intermediates, such as citrate, succinate, and 2-ketoglutarate, were found to be decreased in HFD group mice (**Table 1** **,** **Table 2** **and** **Table 3**). These findings indicated that TCA cycle activity and the homeostasis of energy metabolism were both affected by HFD feeding. In the GA-treated group, the levels of metabolites related to anaerobic (lactate) and aerobic glycolysis, such as pyruvate and lactate, show a recovering trend compared to those in the HFD group mice, while other metabolites, such as citrate, succinate and 2-ketoglutarate, exhibit no manifest change (**Table 1** **,** **Table 2** **and** **Table 3**). These results demonstrated that GA treatment does seems to have an effect in NAFLD mice and that this occurs via an improvement in glycolysis rather than via changes in the metabolism associated with the TCA cycle.

#### Taurine and bile acid metabolism

Taurine is a most abundant amino acid-like compound that is involved in many important physiological processes, including stabilization of the cellular plasma membrane, osmorregulation, anti-oxidative effects, and hepatic detoxification [58]. In the liver, either taurine or glycine can be conjugated with hepatic bile acids in order to allow excretion into bile [58]. Previous studies have been suggested that urinary taurine is a non-invasive biomarker for various types of liver damage and reflect changes in protein metabolism [59]–[61]. This increase in urine taurine is a result of leakage of taurine from damage hepatocytes, and an inhibition of protein synthesis by hepatotoxicants, which has been shown to increase urinary taurine excretion in rats [59]–[61]. Furthermore, a recent study has also proposed the preventive and therapeutic effects of dietary taurine supplementation as a treatment for alcoholic steatohepatitis and NAFLD [58]. In the current study, increased amounts of urinary taurine and glycine were detected in the HFD-fed mice (**Table 2** **and** **Table 3**), which indicates that HFD feeding not only disturbs bile acid metabolism in the liver, but also leads to hepatocyte destruction. The urine levels of taurine and glycine found in the samples from GA-treated mice were significantly reduced compared with those from the urine of animals fed the HFD (**Table 2** **and** **Table 3**). These findings indicate that GA treatment reversed the changes in urine taurine and glycine and that this probably occurs through the hepatoprotective effect of GA, whereby there is an amelioration of disordered bile acid metabolism.

#### Amino acids metabolism

The levels of the glucogenic amino acids (alanine, valine, glutamine, arginine, glycine) as well as those of the ketogenic and glucogenic amino acids (isoleucine, tyrosine, phenylalanine) were decreased in the HFD group mice compared with the levels in the normal group mice (**Table 1** **and** **Table 3**). Our results are consistent with previous observation whereby a HFD cause an impairment of insulin signaling and the ability of insulin to regulate gluconeogenesis [57]. The reduction in glucogenic amino acids may reflect the promotion of gluconeogenesis, which is observed when there is an increased level of glucose (**Figure 1** **and** **Table 3** **)**. Additionally, it is now well established that skeletal muscle is the principle storage target site for insulin-stimulated glucose uptake. In the IR state, skeletal muscle cells shows impaired insulin activity with respect to both glucose transport and intracellular glucose metabolism [62]. As a result of these changes, the aromatic amino acids (tyrosine, phenylalanine), the BCAAs (valine, isoleucine, leucine), as well as glutamate and glutamine are fed into TCA cycle in order to produce ATP and energy for the skeletal muscle. Interestingly, the levels of amino acids in GA treatment mice was found to show a tendency towards recovery compared with similar levels in the HFD-fed mice (**Table 1** **and** **Table 3**). These findings demonstrated that GA treatment is able to ameliorate the IR state in the peripheral tissues, and that this then affect the pathways associated with amino acids metabolism.

Glutamine and glutamate are both precursors of glutathione, the first line of defense against free radicals in the liver [63]. A clinical investigation has indicated reduced plasma glutamate is able to act as a biomarker for septic shock patients with acute liver dysfunction [64]. In the present study, we noted that there was a significantly decreased level of serum glutamate and glutamine in the HFD-fed mice (**Table 1** **and** **Table 3**), which probably reflects the presence of the HFD-induced promotion of oxidative stress [65]. GA treatment reversed this significant decrease in the level of glutathione-associated amino acids (**Table 1** **and** **Table 3**). In agreement with our findings, a previous study has also shown that GA enhances the level of glutathione in the liver and reduces oxidative stress in HFD-fed rats [10]. These results suggest that the hepatic protective effect of GA in this area of metabolism is probable due to GA’s anti-oxidative activity.

#### Choline metabolism

There are three different metabolic pathways involved in choline metabolism [37] (**Figure 7**): In the first, the oxidized choline is excreted as betaine in the urine, which ultimately leads to the production of creatine and creatinine. In the second, choline is converted to methylamine (TMA, TMAO and DMA) by the gut microbiota. While in the third, choline is phosphorylated by choline kinase to generate PC.

Betaine is an essential osmoregulatory compound and an important cofactor in methylation during the methionine-homocysteine cycle [22], [66]. A previous study has shown that betaine insufficiency is associated with metabolic syndrome, lipid disorders and, diabetes as well as playing a crucial role in vascular and other diseases [66]. Moreover, betaine administration was found to significantly improve IR in a NAFLD animal model [67], whereas betaine treatment of NASH patient was found to decrease their steatosis indices [68]. However, the mechanisms by which betaine ameliorates hepatic steatosis have not been fully understood. In this study, compared with the normal diet group mice, a decreased level of betaine was observed in the HFD-fed mice (**Table 1** **and** **Table 3**), which seems to reflect the HFD-induced promotion of oxidative stress; this then inhibits methylamine metabolism, which might be implicated in the pathogenesis of fatty liver. Our analysis shows that the levels of betaine in the GA-treated mice were found to recover and return towards those found in the normal group mice (**Table 1** **and** **Table 3**). Therefore, we suggested that the methylamine metabolism pathway might be another treatment target of GA.

Methylamine-associated metabolites, such as TMA, TMAO and DMA, are the products of the metabolism of choline by gut microbiota [37]. Consistent with a recent study [69], lower levels of TMA and DMA were found in both the HFD and treatment groups (**Table 2** **and** **Table 3**); these changes are most probably due to a dietary effect. On other hand, when the HFD and GA treatment groups were compared, the HFD group mice were found to have higher levels of methylamine-associated metabolites (**Table 2** **and** **Table 3**), which suggests that GA is able to reduce the elevation in gut microflora choline metabolism present in HFD fed mice to a similar level to that of the low-choline diet condition and thereby reduce the induction of severe hepatic steatosis [37]. In addition, it is likely that other changes in gut microbiota-related metabolites in the HFD-fed mice, including changes in hippurate and various short chain organic acids (acetate, butyrate, and isobutyrate), are also associated with the changes to gut microbiota (**Table 1** **,** **Table 2** **and** **Table 3** **)**. Our findings are consistent with previous studies [70], [71] showing that short chain organic acids are produced by gut bacterial fermentation of carbohydrates such as cellulose and resistant starches. In present study, GA treatment not only reversed elevated choline metabolism, but also seemed to improve disorders in gut microbiota-related metabolites (**Table 1** **,** **Table 2** **and** **Table 3** **)**; this supports the hypothesis that the gut microbiota are a probable target for GA treatment [72]. These findings confirm those reported by Bialonska et al. [73] wherein GA-rich fruits seem to cause an enhancement in the growth of probiotic bacteria. In future studies, how GA affected gut microbiota should be further investigated using a metagenomic approach.

#### Nicotinate and nicotinamide metabolism

Nicotinamide, also known as niacinamide and nicotinic acid amide, is the amide derivative of nicotinic acid (vitamin B_3_/niacin) [74]. Nicotinamide is the precursor for two cofactors, NAD^+^ (nicotinamide adenine dinucleotide) and NADP^+^ (nicotinamide adenine dinucleotide phosphate), which both play essential roles in redox reactions [75]. Through the nicotinamide metabolic pathway, nicotinamide is able to be oxidized to nicotinamide N-oxide, methy1ated to 1-methy1nicotinamide, or methy1ated to trigonelline, all of which can be excreted into urine [74]. 1-Methy1nicotinamide has been suggested as a urine biomarker of peroxisome proliferation in rats [76]. Compared with the normal group, there were relatively decreased levels of nicotinamide and trigonelline observed in HFD-induced NAFLD mice compared to control mice, together with increased levels of 1-methylnicotinamide and nicotinamide-N-oxide (**Table 2** **,** **Table 3**). These results indicate that HFD feeding seems to alter nicotinate and nicotinamide metabolic pathway. However, the levels of nicotinamide related metabolites in the GA-treated mice did not show a significant recovery towards the levels from in the control mice. Nonetheless, there was a trend towards recovery compared with the levels found in the HFD group mice (**Table 2** **,** **Table 3**). This implies that GA treatment does not have a primary effect on the metabolic pathways involved in nicotinate and nicotinamide metabolism, but it is possible that there is a secondary effect.

## Conclusions

On the basis of the changes in metabolites identified in this study, a series of metabolic pathway that seem to be associated with HFD-induced hepatosteatosis are proposed in **Figure 7**. These results are based on a 16 weeks HFD feeding regimen that caused metabolome changes in the overall metabolic pathways of a NAFLD mice model. Interestingly, it is important to note that the disturbed metabolic pathways are able to be partially reversed by GA treatment. Our results indicate that the targets of GA treatment are lipid metabolism and ketogenesis, glycolysis, amino acids metabolism, choline metabolism, and gut-microbiota metabolism. These changes are probably useful as novel preventive action biomarkers and also can be used to explore the mechanism by which GA treatment restore normal metabolism. Finally, the current investigation provides further evidence in support of GA as natural dietary compound that is able to ameliorate NAFLD and other metabolic disorders.

## Supporting Information

Figure S1 **The flowchart and study design of the experiment in this paper.** (A) Animal experiment. (B) The NMR metabolomics analysis.
(TIF)

Figure S2 **Gallic acid reduces (A) the body weight but not affect (B) food intake of mice with hepatic steatosis induced by feeding a high fat-diet.**
(TIF)

Figure S3 **PCA results for normal chow diet and high fat diet-fed mice derived from the ^1^H NMR results (A) CPMG spectra of serum, (B) NOESY spectra of serum, (C) BPP-LED spectra of serum, and (D) NOESY spectra of urine.** The continuous-line ellipse indicates the 95% confidence region for Hotelling’s T2 statistics. Nor, normal group; HFD, high fat diet group.
(TIF)

Figure S4 **PLS-DA scatter score plots (left) for serum and urine samples and permutation test plots (200 permutations, right).** (A) and (B) are CPMG spectra of serum. (C) and (D) are NOESY spectra of serum. (E) and (F) BPP-LED spectra of serum. (G) and (H) NOESY spectra of urine. The Y-axis shows the R^2^Y (green filled dots) and Q^2^Y (blue filled square) values of every model, whereas the X-axis indicates the correlation coefficient between original and permuted data response [77]. The Y intercepts of plot for the R^2^Y and Q^2^Y in every model are expressed as numbers. Nor, normal group; HFD, high fat diet group.
(TIF)

Table S1 **The origin of calories from different diets.**
(DOCX)

Table S2 **NMR signals assignment of serum metabolites in mice.**
(DOCX)

Table S3 **NMR signals assignment of urine metabolites in mice.**
(DOCX)

Table S4 **NMR signals assignment of lipid-soluble metabolites of liver in mice.**
(DOCX)

File S1 **The data sheet of normal diet and HFD.**
(DOCX)
