# Supplementary figures and images for: Gallic Acid Ameliorated Impaired Glucose and Lipid Homeostasis in High Fat Diet-Induced NAFLD Mice
Source: PLoS One. 2014 Jun 11;9(6):e96969. doi: 10.1371/journal.pone.0096969 (PMC4053315; doi:10.1371/journal.pone.0096969)

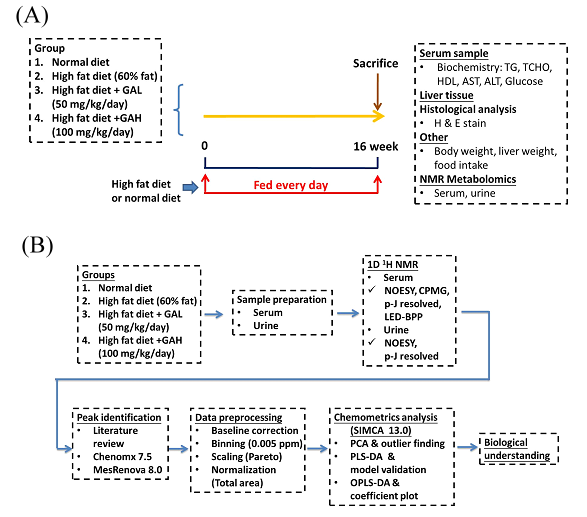

Supplement: Figure S1 — The flowchart and study design of the experiment in this paper. (A) Animal experiment. (B) The NMR metabolomics analysis. (TIF) [file pone.0096969.s001.tif]

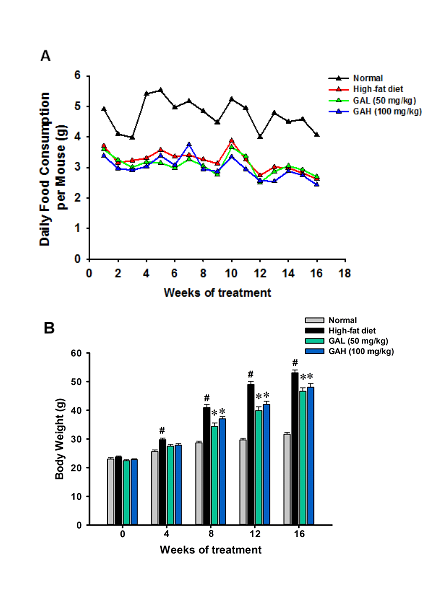

Supplement: Figure S2 — Gallic acid reduces (A) the body weight but not affect (B) food intake of mice with hepatic steatosis induced by feeding a high fat-diet. (TIF) [file pone.0096969.s002.tif]

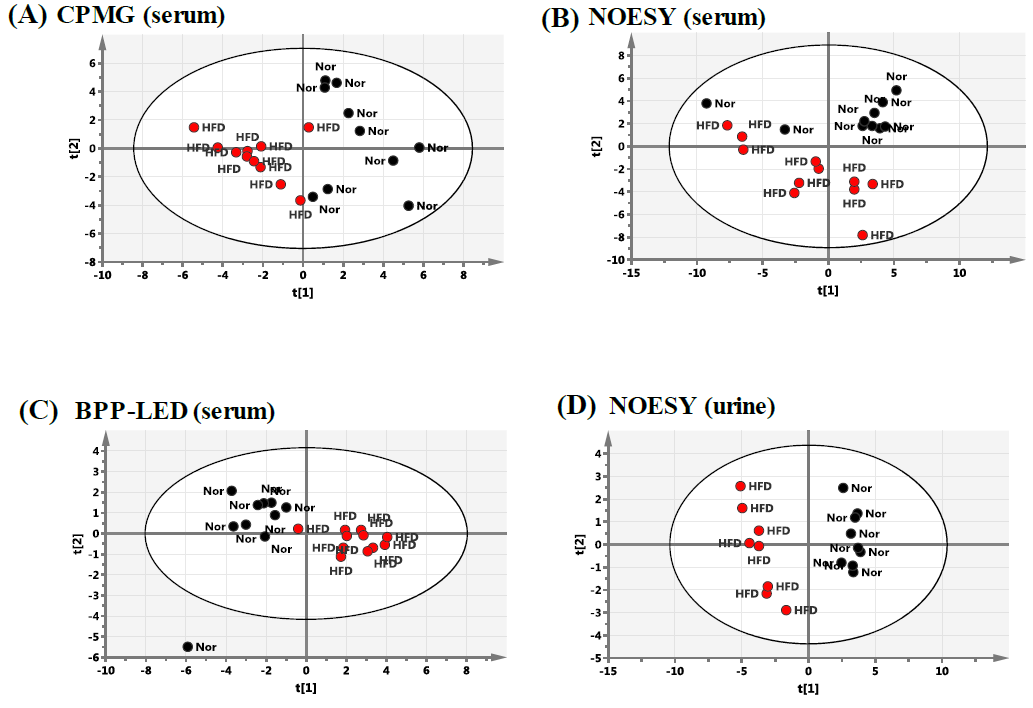

Supplement: Figure S3 — PCA results for normal chow diet and high fat diet-fed mice derived from the 1H NMR results (A) CPMG spectra of serum, (B) NOESY spectra of serum, (C) BPP-LED spectra of serum, and (D) NOESY spectra of urine. The continuous-line ellipse indicates the 95% confidence region for Hotelling’s T2 statistics. Nor, normal group; HFD, high fat diet group. (TIF) [file pone.0096969.s003.tif]

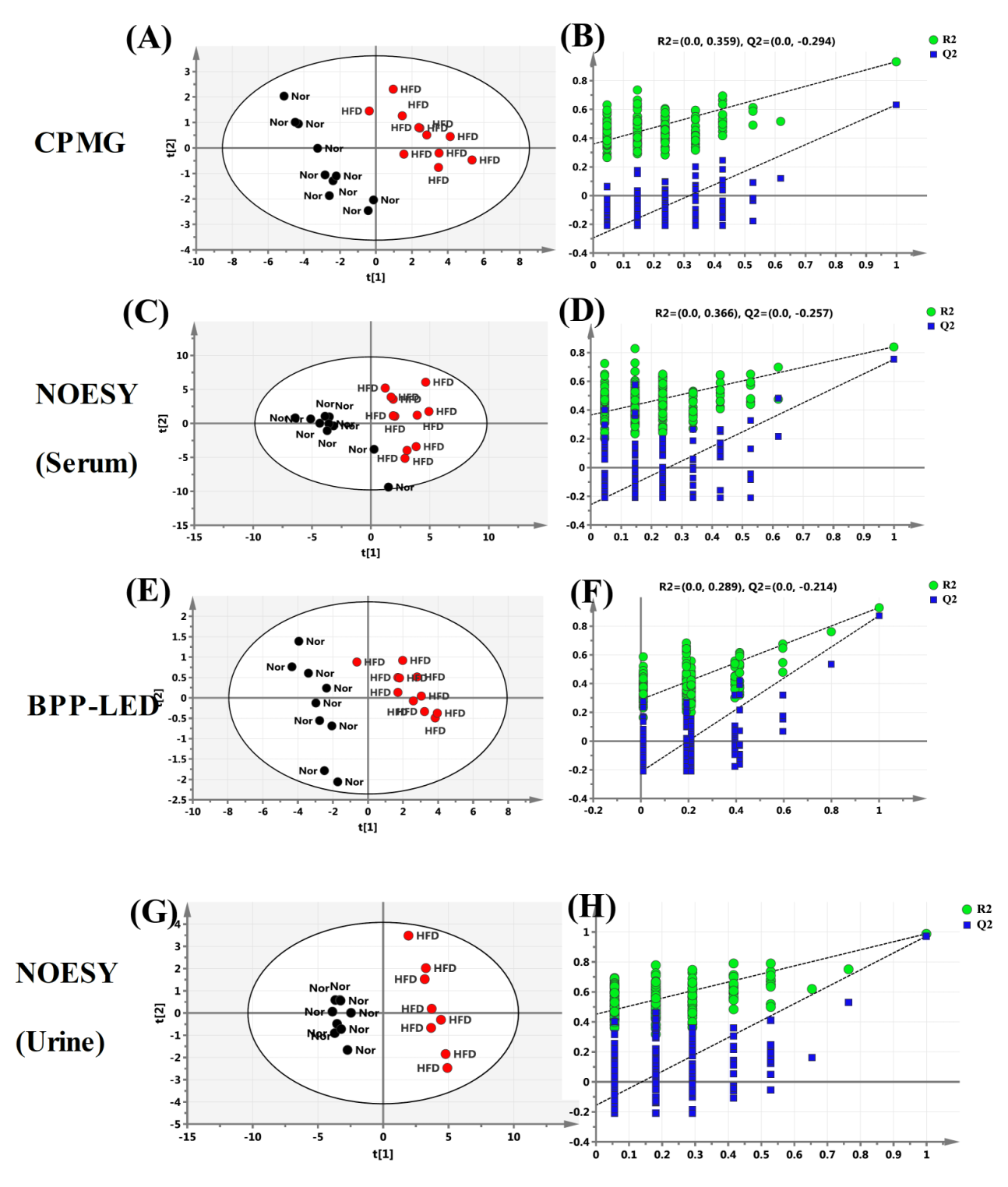

Supplement: Figure S4 — PLS-DA scatter score plots (left) for serum and urine samples and permutation test plots (200 permutations, right). (A) and (B) are CPMG spectra of serum. (C) and (D) are NOESY spectra of serum. (E) and (F) BPP-LED spectra of serum. (G) and (H) NOESY spectra of urine. The Y-axis shows the R2Y (green filled dots) and Q2Y (blue filled square) values of every model, whereas the X-axis indicates the correlation coefficient between original and permuted data response [77]. The Y intercepts of plot for the R2Y and Q2Y in every model are expressed as numbers. Nor, normal group; HFD, high fat diet group. (TIF) [file pone.0096969.s004.tif]

**Others:**

**1. The data sheet of normal diet.**


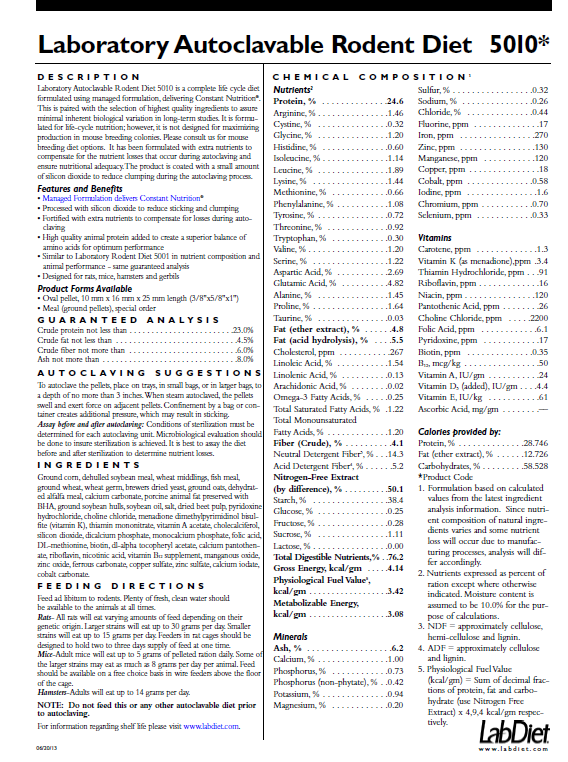


**2. The data sheet of High-fat diet.**


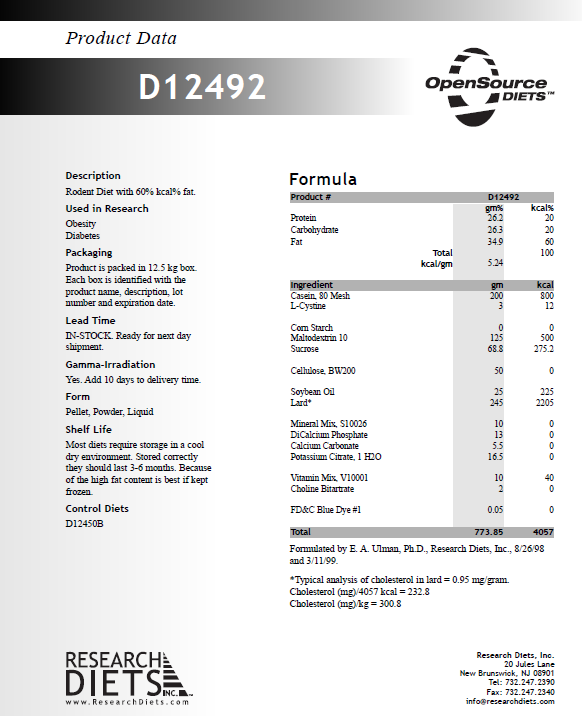

Supplement: File S1 — The data sheet of normal diet and HFD. (DOCX) [file pone.0096969.s009.docx]
