# Supplementary material for: Gallic Acid Ameliorated Impaired Glucose and Lipid Homeostasis in High Fat Diet-Induced NAFLD Mice
Source: PLoS One. 2014 Jun 11;9(6):e96969. doi: 10.1371/journal.pone.0096969 (PMC4053315; doi:10.1371/journal.pone.0096969)
Supplement: Table S1 — The origin of calories from different diets. (DOCX) [file pone.0096969.s005.docx]

**Table S1 The origin of calories from different diets**

| **Diet** | **Carbohydrate (%)** | **Fat (%)** | **Protein (%)** |
| --- | --- | --- | --- |
| Normal diet  (4.14 kcal/gm) | 58.5 | 12.7 | 28.8 |
| High fat diet  (5.24 kcal/gm) | 20 | 60 | 20 |
