# Supplementary material for: Gallic Acid Ameliorated Impaired Glucose and Lipid Homeostasis in High Fat Diet-Induced NAFLD Mice
Source: PLoS One. 2014 Jun 11;9(6):e96969. doi: 10.1371/journal.pone.0096969 (PMC4053315; doi:10.1371/journal.pone.0096969)
Supplement: Table S2 — NMR signals assignment of serum metabolites in mice. (DOCX) [file pone.0096969.s006.docx]

**Table S2 NMR signals assignment of serum metabolites in mice.**

| **No.** | **Metabolites** | **Assignments** | **δ ^1^H (ppm), coupling constant, multiplicity ^a^** | **Observed** |
| --- | --- | --- | --- | --- |
| 1 | HDL | CH_3_(CH_2_)_n_ | 0.86 (m) | CPMG, NOESY, BPP-LED, p-J resolved |
| 2 | LDL | CH_3_(CH_2_)_n_ | 0.88 (m) | CPMG, NOESY, BPP-LED, p-J resolved |
| 3 | Isoleucine | γCH_3_, δCH_3_ | 1.01^※^(d, J=7.0 Hz), 0.94(t) | CPMG, NOESY, p-J resolved |
| 4 | leucine | δCH_3_ + δ'CH_3_ | 0.97 (t) | CPMG, NOESY, p-J resolved |
| 5 | Valine | γCH_3_, γ'CH_3_ | 0.99(d, J=7.0 Hz), 1.04^※^(d, J=7.0 Hz) | CPMG, NOESY, p-J resolved |
| 6 | Isobutyrate | βCH_3_ | 1.14(d) | CPMG |
| 7 | 3-hydroxybutyrate | γCH_3_ | 1.20 (d, J=7.0 Hz) | CPMG, NOESY, p-J resolved |
| 8 | Lipid (triglycerides and fatty acid) | CH_3_(CH_2_)_n_, (CH_2_)_n_  CH_2_─C=C, CH_2_ CH_2_─C=O | 1.24 (m), 1.28^※^(m),  2.04 (m), 2.24 (m) | CPMG, BPP-LED |
| 9 | Lactate | αCH, βCH_3_ | 4.11^※^(q), 1.33 (d, J=7.0 Hz) | CPMG, NOESY, BPP-LED, p-J resolved |
| 10 | Alanine | αCH, βCH_3_ | 1.47^※^(d, J=7.0 Hz), 3.77 (q) | CPMG, NOESY, p-J resolved |
| 11 | Lysine | βCH_2_, γCH_2_, δCH_2,_ εCH_2_ | 1.73 (m), 1.89 (m), 1.47 (m), 3.03^※^(t) | CPMG, NOESY, p-J resolved |
| 12 | Arginine | αCH, βCH_2_, γCH_2_, δCH_2_ | 3.76 (t), 1.89 (m) 1.66 (m), 3.25^※^(t) | CPMG, NOESY, p-J resolved |
| 13 | Ornithine | βCH_2_, γCH_2_, δCH_2_ | 1.95(m), 1.82 (m), 3.06^※^(t) | CPMG, NOESY |
| 14 | Acetate | CH_2_─C=O | 1.92 (s) | CPMG, NOESY, p-J resolved |
| 15 | Proline | αCH, βCH_2_, γCH_2_, δCH_2_ | 4.12 (m), 2.07 (m), 2.00^※^(m), 3.35(t) | CPMG, NOESY |
| 16 | N-acetyl-glycoprotein | CH_3_─C=O | 2.04 (s) | CPMG, NOESY, BPP-LED |
| 17 | O-acetyl-glycoprotein | CH_3_ | 2.14 (s) | CPMG, NOESY, BPP-LED |
| 18 | Glutamate | βCH_2_, γCH_2_ | 2.08 (m), 2.36^※^(m) | CPMG, NOESY |
| 19 | Glutamine | αCH, βCH_2_, γCH_2_ | 3.68 (t), 2.15 (m), 2.45^※^(m) | CPMG, NOESY |
| 20 | Acetoacetate | CH_3_, CH_2_ | 2.29^※^(s), 3.49(s) | CPMG, NOESY, p-J resolved |
| 21 | Pyruvate | βCH_3_ | 2.37 (s) | CPMG, NOESY, p-J resolved |
| 22 | Succinate | α, βCH_2_ | 2.40 (s) | CPMG, NOESY, p-J resolved |
| 23 | Citrate | half CH_2,_ half CH_2_ | 2.53 (d, J=15.8 Hz), 2.69 (d, J=15.8 Hz) | CPMG, NOESY, p-J resolved |
| 24 | Polyunsaturated fatty acid (PUFA) | =C─CH_2_─C= | 2.75(m) | CPMG, NOESY, BPP-LED |
| 25 | Albumin | Lysyl- CH_2_ | 3.02 | BPP-LED |
| 26 | Creatine | N- CH_3_, CH_2_ | 3.04^※^(s), 3.93 (s) | CPMG, NOESY, p-J resolved |
| 27 | Creatinine | 5-CH, 4,6-CH, 2-CH | 3.05 (s), 4.06^※^(s) | CPMG, NOESY, p-J resolved |
| 28 | Choline | N(CH_3_)_3_ | 3.21 (s) | CPMG, NOESY, p-J resolved |
| 29 | Phosphocholine | N(CH_3_)_3_ | 3.23 (s) | CPMG, NOESY, p-J resolved, BPP-LED |
| 30 | TMAO | CH_3_ | 3.26 (s) | CPMG, NOESY, p-J resolved |
| 31 | Taurine | N─CH_2_,S─CH_2_, | 3.26 (t), 3.40^※^(t) | CPMG, NOESY, p-J resolved |
| 32 | Betaine | CH_3_, CH_2_ | 3.27^※^(s), 3.90 (s) | CPMG, NOESY, p-J resolved |
| 33 | α-Glucose | H1, H2, H3, H4, H5, H6, H6’ | 4.65 (d, J=8.0 Hz), 3.24^※^(dd), 3.49 (t) 3.42(t), 3.47 (m), 3.90 (dd), 3.72 (dd) | CPMG, NOESY, p-J resolved, BPP-LED |
| 34 | β-Glucose | H1, H2, H3, H4, H5, H6, H6’ | 5.24^※^(d, J=3.7 Hz), 3.53 (dd), 3.69 (t), 3.39 (t), 3.81 (m), 3.71(dd), 3.73 (dd) | CPMG, NOESY, p-J resolved, BPP-LED |
| 35 | Glycine | CH_2_ | 3.56 (s) | CPMG, NOESY, p-J resolved |
| 36 | Unsaturated fatty acid (UFA) | ─CH=CH─ | 5.29 (m) | CPMG, NOESY, BPP-LED |
| 37 | Fumarate | CH | 6.52 (s) | CPMG, p-J resolved |
| 38 | Tyrosine | CH, CH | 6.90^※^(d, J=8.0), 7.20 (d, J=8.0) | CPMG, NOESY, p-J resolved |
| 39 | 1-Methylhistidine | 4-CH, 2-CH | 7.06^※^(s), 7.77 (s) | CPMG |
| 40 | Phenylalanine | 2, 6-CH, 3,5-CH, 4-CH | 7.33^※^(m), 7.38 (m), 7.42(m) | CPMG, NOESY |
| 41 | Formate | CH | 8.46 (s) | CPMG, p-J resolved |

^a^ Peaks observed as singlet (s), doublet (d), triplet (t), quartet (q), multiplet (m), or broad (b).

HDL: high-density lipoprotein；LDL: -density lipoprotein；TMAO: trimethylamine N-oxide
