# Supplementary material for: Gallic Acid Ameliorated Impaired Glucose and Lipid Homeostasis in High Fat Diet-Induced NAFLD Mice
Source: PLoS One. 2014 Jun 11;9(6):e96969. doi: 10.1371/journal.pone.0096969 (PMC4053315; doi:10.1371/journal.pone.0096969)
Supplement: Table S3 — NMR signals assignment of urine metabolites in mice. (DOCX) [file pone.0096969.s007.docx]

**Table S**3 **NMR signals assignment of urine metabolites in mice.**

| **No.** | **Metabolites** | **Assignments** | **δ 1H (ppm), coupling constant, multiplicity ^a^** | **Observed** |
| --- | --- | --- | --- | --- |
| 3 | Isoleucine | γCH_3_, δCH_3_ | 1.01^※^(d, J=7.0 Hz), 0.94(t) | NOESY, p-J resolved |
| 4 | leucine | δCH_3_ + δ'CH_3_ | 0.97 (t) | NOESY, p-J resolved |
| 5 | Valine | γCH_3_, γ'CH_3_ | 0.99(d, J=7.0 Hz), 1.05^※^(d, J=7.0 Hz) | NOESY, p-J resolved |
| 9 | Lactate | αCH, βCH_3_ | 4.11 (q), 1.33^※^(d, J=7.0 Hz) | NOESY, p-J resolved |
| 11 | Lysine | βCH_2_, γCH_2_, δCH_2_ | 1.73^※^(m), 1.89 (m), 1.47 (m) | NOESY, p-J resolved |
| 12 | Arginine | αCH, βCH_2_, γCH_2_, δCH_2_ | 3.76 (t), 1.89^※^(m) 1.63 (m), 3.25 (t) | NOESY, p-J resolved |
| 14 | Acetate | CH_2_─C=O | 1.92 (s) | NOESY, p-J resolved |
| 16 | N-acetyl-group | CH_3_─C=O | 2.04 (s) | NOESY, p-J resolved |
| 20 | Acetoacetate | CH_3_, CH_2_ | 2.29^※^(s), 3.49(s) | NOESY, p-J resolved |
| 21 | Pyruvate | βCH_3_ | 2.37 (s) | NOESY, p-J resolved |
| 22 | Succinate | α, βCH_2_ | 2.41 (s) | NOESY, p-J resolved |
| 23 | Citrate | half CH_2_, half CH_2_ | 2.55^※^(d, J=15.8 Hz), 2.69 (d, J=15.8 Hz) | NOESY, p-J resolved |
| 26 | Creatine | N- CH_3_, CH_2_ | 3.04^※^(s), 3.93 (s) | NOESY, p-J resolved |
| 27 | Creatinine | 5-CH, 4,6-CH, 2-CH | 3.05 (s), 4.06^※^(s) | NOESY, p-J resolved |
| 28 | Choline | N(CH_3_)_3_ | 3.21 (s) | NOESY, p-J resolved |
| 31 | Taurine | N─CH_2_,S─CH_2_, | 3.27 (t), 3.43^※^(t) | NOESY, p-J resolved |
| 35 | Glycine | CH_2_ | 3.55 (s) | NOESY, p-J resolved |
| 37 | Fumarate | CH | 6.52 (s) | NOESY, p-J resolved |
| 41 | Formate | CH | 8.46 (s) | NOESY, p-J resolved |
| 42 | α-Keto-β-methylvaleric acid (3-Methyl-2-oxovalerate) | CH_3_, CH_3_, CH_2_, CH_2_ | 0.88^※^(t), 1.10 (d) 1.72 (m), 1.46 (m) | NOESY, p-J resolved |
| 43 | Butyrate | CH_3_, CH_2_, CH_2_ | 0.90^※^(t) 1.58 (m), 2.14 (t) | NOESY, p-J resolved |
| 44 | Isovalerate | CH_3_, CH, CH_2_ | 0.92^※^(d), 1.94 (m), 2.04 (d) | NOESY, p-J resolved |
| 45 | 2-oxoisocaproate | δCH_3_, CH, γCH_2_ | 0.94 (d), 2.08 (m), 2.61 (m) | NOESY, p-J resolved |
| 46 | 3-hydroxy-isovalerate | CH_3_ | 1.21 (s) | NOESY, p-J resolved |
| 47 | Methylmalonate | CH_3_ | 1.24 (d) | NOESY |
| 48 | 2-hydroxyisobutyrate | CH_3_ | 1.36 (s) | NOESY, p-J resolved |
| 49 | Acetamide | CH_3_ | 2.00 (s) | NOESY, p-J resolved |
| 50 | 2-oxoglutaric acid | CH_2,_ CH_2_ | 2.44 (t, J=6.89 Hz), 3.01 (t, J=6.89 Hz) | NOESY, p-J resolved |
| 51 | Methylamine | CH_3_ | 2.60 (s) | NOESY, p-J resolved |
| 52 | Dimethylamine (DMA) | CH_3_ | 2.72 (s) | NOESY, p-J resolved |
| 53 | Trimethylamine (TMA) | CH_3_ | 2.89 (s) | NOESY, p-J resolved |
| 54 | Cis-aconitate | CH_2_, CH | 3.11 (s), 5.63 (s) | NOESY, p-J resolved |
| 55 | Carnitine | N(CH_3_)_3_ | 3.23 (s) | NOESY, p-J resolved |
| 56 | TMAO | CH_3_ | 3.27 (s) | NOESY, p-J resolved |
| 57 | Guanidoacetate | CH_2_ | 3.80 (s) | NOESY, p-J resolved |
| 58 | Trans-aconitate | =CH_,_ CH_2_ | 6.59(s), 3.45 (s) | NOESY, p-J resolved |
| 59 | Phenylacetate | 4-CH, 3,5-CH, CH_2_ | 7.31 (t), 7.38 (t), 3.54^※^(s) | NOESY, |
| 60 | Glucose (α & β form) | H1, H1 H6 | 4.65 (d, J=8.0 Hz), 5.24 (d, J=3.7 Hz) 3.90^※^(dd) | NOESY, p-J resolved |
| 61 | Hippurate | CH_2_, NH, H2 & H6, H3 & H5, H4 | 3.95 (d), 8.55(s), 7.84 (d), 7.56(t), 7.64^※^(t) | NOESY, p-J resolved |
| 62 | Trigonelline | CH_3_, 5-CH, 4,6-CH, 2-CH | 4.44 (s), 8.08 (t), 8.84 (t), 9.13 (s) | NOESY |
| 63 | 1-Methylnicotinamide | CH_3_, H_5_, H_4_, H_6_, H_2_ | 4.47 (s), 8.17 (t), 8.90 (d), 8.97 (d), 9.28 (s) | NOESY |
| 64 | Cis-aconitic acid | CH, CH_2_ | 5.69 (s), 3.11 (s) | NOESY, p-J resolved |
| 65 | Urea | NH_2_ | 5.80 (br) | NOESY |
| 66 | Allantoate (allantoin) | CH | 5.40 (s) 6.03 (s) | NOESY |
| 67 | Carnosine | CH, CH | 7.08 (s), 8.12 (s) | NOESY |
| 68 | 3-Indoxylsulfate | 5-CH, 6-CH , 2-CH, 7-CH, 4-CH | 7.21 (t), 7.27 (t), 7.36 (s), 7.50 (d), 7.71^※^(d) | NOESY, , p-J resolved |
| 69 | Benzoate | Ring-CH | 7.83 (m), 7.47^※^(t), 7.55 (m) | NOESY |
| 70 | Nicotinamide N-oxide | Ring-CH | 7.74 (t, dd), 8.09 (d), 8.49 (d, J=6.8 Hz), 8.74^※^(t) | NOESY |
| 71 | Phenylalanine | Ring-CH | 7.42^※^(m), 7.33(m), 7.35 (m) | NOESY |
| 72 | Uracil | CH, CH | 7.51^※^(d), 5.90 (d) | NOESY |
| 73 | Niacinamide | Ring-CH | 8.94 (br), 8.70 (d) | NOESY |

^a^ Peaks observed as singlet (s), doublet (d), triplet (t), quartet (q), multiplet (m), or broad (b)
