# Supplementary material for: Gallic Acid Ameliorated Impaired Glucose and Lipid Homeostasis in High Fat Diet-Induced NAFLD Mice
Source: PLoS One. 2014 Jun 11;9(6):e96969. doi: 10.1371/journal.pone.0096969 (PMC4053315; doi:10.1371/journal.pone.0096969)
Supplement: Table S4 — NMR signals assignment of lipid-soluble metabolites of liver in mice. (DOCX) [file pone.0096969.s008.docx]

**Table S4 NMR signals assignment of lipid-soluble metabolites of liver in mice.**

| **No.** | **Metabolites** | **Assignments** | **δ 1H (ppm), coupling constant, multiplicity ^a^** | **Observed** |
| --- | --- | --- | --- | --- |
| 74 | Total Cholesterol | C18-H_3_, C26/27-H_3_, C21-H_3_ | 0.68^※^(s), 0.86 (d, J=2.8 Hz), 0.92 (d, J=6.6 Hz) | NOESY |
| 75 | w-6 fatty acids ^b^ | C***H***_3_, C***H***_3_, C***H***_2_, C***H_2_***C=C, C***H_2_***CH_2_COO, C***H_2_***COO, C=CC***H_2_***C=C | 0.88^※^(t), 0.89^※^(t), 1.30 (m), 2.01 (m), 1.61 (m), 2.31 (m), 2.77 (m) | NOESY |
| 76 | w-3 fatty acids ^b^ | C***H***_3_, C***H_2_***C=C, C=CC***H_2_***C=C, C***H_2_***CH_2_COO, C***H_2_***COO | 0.96^※^(t), 2.05 (m), 2.82 (m), 1.61 (m), 2.31 (m) | NOESY |
| 77 | Triglyceride | Glycerol (C1-H^u^) and (C3-H^u^), Glycerol (C1-H^d^) and (C3-H^d^), Glycerol (C2-H) | 4.14^※^(dd, J=6.0, 11.9 Hz), 4.29^※^(dd, J=4.3, 11.9 Hz), 5.26 (m) | NOESY |

^a^ Peaks observed as singlet (s), doublet (d), triplet (t), quartet (q), multiplet (m), or broad (b)

^b^ The PUFA-to-MUFA ratio was calculated from the spectral regions at δ 5.29−5.44 for UFA (-C***H***=C***H***-), δ 2.73−2.88 for PUFA (-C=C-C***H_2_***-C=C-) and δ 0.81−0.93 for methyl groups of all fatty acids (-CH_3_).
